# Widespread associations between trait conscientiousness and thickness of brain cortical regions

**DOI:** 10.1016/j.neuroimage.2018.04.033

**Published:** 2018-08-01

**Authors:** Gary J. Lewis, David Alexander Dickie, Simon R. Cox, Sherif Karama, Alan C. Evans, John M. Starr, Mark E. Bastin, Joanna M. Wardlaw, Ian J. Deary

**Affiliations:** a Department of Psychology, Royal Holloway, University of London, Egham, Surrey, TW20 0EX, UK; b Scottish Imaging Network, A Platform for Scientific Excellence (SINAPSE) Collaboration, Glasgow, UK; c Brain Research Imaging Centre, Neuroimaging Sciences, Centre for Clinical Brain Sciences, University of Edinburgh, Western General Hospital, Edinburgh, EH4 2XU, UK; d Department of Psychology, University of Edinburgh, 7 George Square, Edinburgh, EH8 9JZ, UK; e Centre for Cognitive Ageing and Cognitive Epidemiology, University of Edinburgh, 7 George Square, Edinburgh, EH8 9JZ, UK; f Montreal Neurological Institute and Hospital and Douglas Mental Health University Institute (S.K.), McGill University, Canada; g Alzheimer Scotland Dementia Research Centre, University of Edinburgh, 7 George Square, Edinburgh, EH8 9JZ, UK

**Keywords:** Personality, Conscientiousness, Cortical thickness, Brain, Neuroanatomy, Allostatic load

## Abstract

The neural correlates of human personality have been of longstanding interest; however, most studies in the field have relied on modest sample sizes and few replicable results have been reported to date. We investigated relationships between personality and brain gray matter in a sample of generally healthy, older (mean age 73 years) adults from Scotland drawn from the Lothian Birth Cohort 1936. Participants (N = 578) completed a brain MRI scan and self-reported Big Five personality trait measures. Conscientiousness trait scores were positively related to brain cortical thickness in a range of regions, including bilateral parahippocampal gyrus, bilateral fusiform gyrus, left cingulate gyrus, right medial orbitofrontal cortex, and left dorsomedial prefrontal cortex. These associations – most notably in frontal regions – were modestly-to-moderately attenuated by the inclusion of biomarker variables assessing allostatic load and smoking status. None of the other personality traits showed robust associations with brain cortical thickness, nor did we observe any personality trait associations with cortical surface area and gray matter volume. These findings indicate that brain cortical thickness is associated with conscientiousness, perhaps partly accounted for by allostatic load and smoking status.

## Highlights

• We investigated relationships between personality and neuroanatomy.
• Participants (N = 578) completed an MRI scan and Big Five personality trait measures.
• Conscientiousness was positively related to cortical thickness in a range of regions.
• These included: parahippocampal, fusiform, and cingulate gyri, and frontal cortex.
• No other Big Five trait was associated with our brain measures.

## Introduction

Longstanding interest has been directed towards the neural correlates of human personality trait differences (Eysenck, 1967; Gray and McNaughton, 2000; Zuckerman, 2005). In recent years, research has suggested a range of neural links to personality trait scores (e.g. Bjørnebekk et al., 2013; DeYoung et al., 2010; Holmes et al., 2012; Lewis et al., 2016); these studies used a variety of brain scanning techniques such as voxel- and surface-based morphometry, and diffusion tensor MRI. However, this literature is notable for its mixed results, with few studies replicating earlier findings.

Here we sought to contribute to this literature by measuring brain cortical thickness, cortical surface area, and gray matter volume in a relatively large, narrow-age sample (N = 578) of generally healthy older people who underwent an MRI scanning session and also completed a self-report measure of the Big Five personality traits. Our focus on the Big Five personality traits (sometimes referred to as the Five Factor Model) falls in line with the broad-based acknowledgement that these five dimensions collectively describe a substantial portion of response variation across the breadth of personality space (Matthews et al., 2009; McCrae and John, 1992). Recent work has also highlighted the importance of acknowledging the two higher-order ‘meta-traits’ of personality that exist ‘above’ the Big Five: stability (reflecting common variation across agreeableness, conscientiousness, and neuroticism) and plasticity (reflecting common variation across extraversion and openness) (DeYoung, 2006).

Variables from the five personality dimensions and the two meta-traits are predictive of a range of important life outcomes, including disease and mortality (Bogg and Roberts, 2004; Friedman and Booth-Kewley, 1987; Friedman et al., 1993), educational attainment (Rimfeld et al., 2016), socio-economic status (Roberts et al., 2007), relationship outcomes (Karney and Bradbury, 1995; Roberts et al., 2007), and psychopathology (DeYoung et al., 2008; Kotov et al., 2010), highlighting the importance of achieving a deeper understanding of their neurobiological correlates. We next provide an overview of the personality neuroscience literature to date – with a specific focus on findings concerning cortical thickness, cortical surface area, and gray matter volume in samples of N ≥ 100 (in order to reflect the larger studies in the field: see Supplementary Table 1 for a broader overview) – before outlining our analytical approach.

### Gray matter and personality: a brief overview

The interest in delineating the neuroanatomical links to human personality trait differences, coupled with substantial advances in brain imaging technology and analysis tools, has led to a large number of studies in this field in recent years. Perhaps the greatest focus has been directed towards neuroticism and extraversion, in line with their consistent links to psychopathology (Kotov et al., 2010). With regard to neuroticism, positive associations have been reported with gray matter volume in bilateral midcingulate gyrus, left middle temporal gyrus (DeYoung et al., 2010: N = 116), bilateral amygdala (Holmes et al., 2012: N = 1050), and with cortical thickness in left supra-marginal gyrus, right superior parietal cortex, right superior temporal cortex, and bilateral superior prefrontal cortex (Riccelli et al., 2017: N = 507). Negative associations have been noted with cortical thickness in left medial prefrontal cortex (Holmes et al., 2012), with surface area in bilateral middle temporal cortex, left rostral middle frontal gyrus, bilateral superior frontal gyrus, left cuneus, right superior parietal cortex, right frontal pole (Riccelli et al., 2017), and with gray matter volume in left middle temporal cortex, left superior temporal cortex, left lateral occipital cortex, and right fusiform gyrus (Riccelli et al., 2017).

Extraversion has also been associated with a range of brain regions. Positive associations have been reported for gray matter volume in right medial orbitofrontal cortex (DeYoung et al., 2010), bilateral amygdala (Lewis et al., 2014: N = 486), and cortical thickness in the left precuneus (Riccelli et al., 2017). Negative associations have been reported for cortical thickness in left inferior frontal gyrus (Bjørnebekk et al., 2013: N = 265), with surface area in right R superior temporal cortex (Riccelli et al., 2017), and with gray matter volume in left superior temporal gyrus and right entorhinal cortex (Riccelli et al., 2017).

Less research has assessed neuroanatomical links to agreeableness, conscientiousness, and openness. Of work that has been conducted to date, agreeableness has been positively associated with gray matter volume in left posterior cingulate and right fusiform gyrus (DeYoung et al., 2010), and negatively associated with surface area in fusiform cortex (Riccelli et al., 2017) and gray matter volume in left superior temporal sulcus (DeYoung et al., 2010) and left middle/superior frontal cortex (Riccelli et al., 2017). Conscientiousness has been positively associated with gray matter volume in left middle frontal gyrus (DeYoung et al., 2010), and cortical thickness in bilateral middle frontal cortex and right precuneus (Riccelli et al., 2017). Negative associations have been reported with surface area in left lateral occipital cortex and right middle temporal cortex, and with gray matter volume in right fusiform gyrus (DeYoung et al., 2010). Finally, of the studies to address openness, positive associations have been reported with surface area in left inferior temporal cortex, right postcentral gyrus, right lateral occipital cortex, right inferior parietal cortex, and with gray matter volume in bilateral inferior temporal cortex and left temporal pole (Riccelli et al., 2017). Negative associations have been noted with cortical thickness in the postcentral gyrus, rostral anterior cingulate cortex, superior prefrontal cortex, inferior parietal cortex, and lateral occipital gyrus (Riccelli et al., 2017).

This brief overview makes clear that a broad range of brain regions have been implicated with Big Five personality traits (also see Supplementary Table 1). However, it is also apparent that mixed results are the norm rather than the exception, with few studies reporting the same brain regions. For example, orbitofrontal cortex has been associated with extraversion in one study (DeYoung et al., 2010), but not in others (e.g. Bjørnebekk et al., 2013; Lewis et al., 2014; Riccelli et al., 2017). And reported links between amygdala and neuroticism (Holmes et al., 2012) have not been observed in independent studies (e.g. Bjørnebekk et al., 2013; DeYoung et al., 2010; Lewis et al., 2014). Moreover, two of the larger studies in the field to date (N = 227; N = 364, respectively) reported no association between gray matter and any of the Big Five personality traits (Liu et al., 2013; Nostro et al., 2017).

A number of factors may explain the mixed findings. Firstly, many of the studies reported to date have relied on relatively modest sample sizes (Mar et al., 2013) and so may contain chance/sample-specific observations. Secondly, a number of the studies in the field have used voxel-based morphometry (VBM). Although this approach is widely used and well-understood (Mechelli et al., 2005), research in recent years has noted that distinct genetic influences underpin cortical thickness and surface area (Panizzon et al., 2009) which are aggregated in VBM, potentially masking important information. Thirdly, recent work has argued that sex differences may play an important role in the relationship between brain structure and personality. Nostro et al. (2017), using a large sample (N = 364: 182 males), reported no neuroanatomical associations with personality when the sexes were aggregated; however, a range of gray matter associations were noted for a males-only sub-set.

### The current study

With these observations in mind we sought to examine the relationship between cortical thickness, cortical surface area, and gray matter volume and personality using a relatively large sample (N = 578) of generally healthy, older adults who completed an MRI session and a self-report Big Five personality measure. These data allowed us to test for neuroanatomical associations with the Big Five traits and the two meta-traits (stability and plasticity). In our core analyses we included a number of covariates: age at scanning in days, sex, intracranial volume, the non-target personality traits, and general intelligence, in line with known links between intelligence and both cortical thickness (Deary et al., 2010) and Big Five traits (Ackerman and Heggestad, 1997; Ashton et al., 2000).

An observation of personality/neuroanatomy correlates would naturally give rise to questions concerning the nature of this relationship. Clearly our cross-sectional data cannot speak to the causal direction of any such links. It is plausible that individual differences in neuroanatomy give rise to personality differences, or vice versa. And these causal perspectives might be mediated or perhaps even confounded by other variables. Potential mediating or confounding factors include allostatic load (i.e. bodily ‘wear and tear’) and smoking status. Both are related to personality traits (Allen and Laborde, 2017; Friedman and Kern, 2014). Moreover, both are associated with brain structure differences. For example, smoking is associated with cortical thinning in the sample on which the current study is based (Karama et al., 2015). And a number of studies have reported that markers of allostatic load, such as cortisol and C-reactive protein levels, are associated with a range of brain regions (e.g. Kremen et al., 2010; Krishnadas et al., 2013). As such, and only for models showing significant links between personality traits and our brain measures, we further examined whether these links were robust to the inclusion of measures of allostatic load and smoking status.

Finally, while we focused our attention primarily on the aggregated sample, we also sought to test for the presence of sex-dependent associations for each of the personality traits in line with recent work reporting sex differences in gray matter/personality associations (Nostro et al., 2017).

As noted above, the previous literature provides limited scope for confirmatory testing given the lack of consistently identified regions of interest. As such, our analyses were exploratory in nature (see Statistical analyses for more details).

## Methods

### Participants

Participants were all members of the Lothian Birth Cohort 1936 (LBC 1936) and all were born in 1936. When initially recruited at mean age 70 years, the LBC1936 comprised 1091 relatively healthy, community-dwelling individuals without dementia who mostly resided in or close to the city of Edinburgh (the Lothian region), Scotland. Most had taken part in the Scottish Mental Survey of 1947 in which they undertook a well-validated mental ability test at school on June the 4th 1947. At recruitment in older age (Wave 1), participants underwent a variety of cognitive, biomedical, and psychosocial testing including self-rating of personality traits. The data from the LBC1936 used in this study were obtained at Wave 2 when the participants (males = 53.6%) had a mean age of about 73 years (M = 72.73 years; SD = 0.72), and when an optional brain MRI scan was included alongside concomitant data collection of cognitive and physical function, personality, and medical history. Bloods were also drawn for genetic and biomarker analysis. Further details of the study are available in the form of a study protocol (Deary et al., 2007), cohort profile (Deary et al., 2012), and brain imaging protocol (Wardlaw et al., 2011).

Participants travelled to the Wellcome Trust Clinical Research Facility in Edinburgh's Western General Hospital for testing. The study was approved by the Lothian (LREC/2003/2/29), Scottish Multicentre (MREC/01/0/56) and Scotland A (07/MRE00/58) Research Ethics Committees. All subjects gave written informed consent which has been kept on file.

### Measures

**Big Five personality traits** were assessed using the validated and well-characterized International Personality Item Pool (IPIP) 50-item inventory (Goldberg, 1999; Gow et al., 2005). Emotional stability (the opposite of neuroticism), extraversion, intellect (similar to openness), agreeableness, and conscientiousness were each indexed by 10 items. Participants self-reported for each item on a 1 (very inaccurate) to 5 (very accurate) scale. Dimensions were scored as the sum of responses (reverse-scoring where appropriate) across the relevant 10 items. Stability and plasticity were operationalized as a mean score from the relevant trait dimensions. Descriptive statistics and inter-correlations between the personality traits, general intelligence, measures of allostatic load, and smoking status are detailed in Supplementary Tables 2 and 3.

**Allostatic load** was assessed using ten biomarkers: three markers of inflammation (fibrinogen, interleukin-6, and C-reactive protein), five metabolic markers (high- and low-density lipoprotein, body mass index, glycated hemoglobin, and triglyceride), and blood pressure (mean sitting systolic and diastolic blood pressure, taken from 6 serial measurements, 3 sitting and 3 standing). Blood samples were taken during participants' physical examination at Wave 2 of testing.

Allostatic load can be characterised by three specific latent factors (loading on indicators reflecting inflammation, metabolic functioning, and blood pressure, respectively), alongside an overarching general latent factor which captures the covariance among the three specific latent factors (Booth et al., 2015). To this end we used factor scores derived from a confirmatory factor model as specified above (i.e. a bifactor model with one general factor and three specific factors) for use as variables in our imaging analyses. A small number (<3%) of participants had missing data on one or more of the allostatic load measures. As such, full-information maximum-likelihood estimation was used, which is considered to be a robust method in such cases (Enders, 2010).

**Smoking status** was assessed as a binary variable: have you ever been a smoker (no/yes)?

**General intelligence** was assessed as the first principal component derived from a cognitive battery that included several sub-tests from the Wechsler Adult Intelligence Scale (Block Design, Matrix Reasoning, Digit Symbol Coding, Symbol Search, and Letter-Number Sequencing), several sub-tests from the Wechsler Memory Scale (Logical Memory: immediate and delayed recall, Verbal Paired Associates: immediate and delayed recall, Digit Span: backward, Spatial Span: forward and backward), the National Adult Reading Test, the Wechsler Test of Adult Reading, verbal fluency, simple and 4-choice reaction time tasks, and an inspection time task. See Deary et al. (2007) for further details of the tests and their administration.

### Brain imaging

All MRI data were acquired using a GE Signa Horizon HDxt 1.5T clinical scanner (General Electric, Milwaukee, WI, USA) equipped with a self-shielding gradient set (33 mTm^−1^ maximum gradient strength) and manufacturer supplied eight-channel phased-array head coil. The scan session comprised a high-resolution whole-brain T1-weighted (T1W) volume sequence acquired in the coronal plane (for full details of the complete LBC1936 MRI protocol, see Wardlaw et al., 2011). In short, the T1W volume scan was acquired with a field of view of 256 × 256 mm^2^, an acquisition matrix of 192 × 192 (zero filled to 256 × 256) and 160 contiguous 1.3-mm thick slices giving a final voxel dimension of 1 × 1 × 1.3 mm^3^. The repetition, echo and inversion times were 10, 4, and 500 ms, respectively. To accurately measure intracranial volume, slices were placed to cover the complete intracranial contents from above the skull vertex to the upper cervical spine below the foramen magnum.

#### Image processing

To obtain vertex-wise cortical thickness, cortical surface area, and gray matter volume measurements for each subject, all T1W volume scans were processed by the CIVET pipeline (version 1.1.12) developed at the Montreal Neurological Institute (http://www.bic.mni.mcgill.ca) for fully automated structural image analysis. The CIVET pipeline processing steps were implemented using the Canadian Brain Imaging Network (http://www.cbrain.mcgill.ca) (Frisoni et al., 2011). Steps include (also see Karama et al., 2009): (1) registering T1W images to a standardized space using an age-specific template; (2) correcting for intensity nonuniformity artifacts (bias field); (3) producing high-resolution hemispheric surfaces with 40962 vertices each; (4) registering surfaces to a high-resolution template to establish inter-subject correspondence of vertices; (5) applying a reverse of step 1 to allow cortical thickness, cortical surface area, and gray matter volume estimations in the native space of each subject; (6) calculating cortical thickness, cortical surface area, and gray matter volume at each vertex using the t-link metric; and (7) smoothing using a 20-mm kernel. In a final step, visual quality control of the native cortical gray and white matter surfaces was implemented to make sure that there were no important aberrations in cortical thickness, cortical surface area, and gray matter volume estimations for a given participant. Raters were blind to each subject's demographic and cognitive characteristics. Participants (approximately 10%) for whom there were clear problems with the brain maps (e.g. ringing or other such artifacts) were removed from further analysis. A total of N = 578 subjects with personality measures passed visual QC of brain maps and were included in this analysis.

### Statistical analyses

SurfStat (http://www.math.mcgill.ca/keith/surfstat), implemented in MATLAB 2014a was used to perform statistical analyses. We used multiple regression to examine each subject's absolute native-space cortical thickness, cortical surface area, and gray matter volume at each vertex against their Big Five personality/meta-trait scores. This was done while accounting for the effects of sex, age at scanning in days (in order to account for any residual age effect), intracranial volume, the other Big Five traits (or stability/plasticity for analyses of the meta-traits), and general intelligence. In order to assess whether or not there were sex differences in any association observed between our brain measures and personality, a sex-by-personality interaction term was also examined. For models showing significant links between personality traits and our brain measures we additionally examined whether these links were robust to the inclusion of measures of allostatic load and smoking status.

Thresholds of significance for the resulting *t*-test values of the regressor coefficients were calculated by taking into account multiple comparisons through false discovery rate (Genovese et al., 2002): this method controls for the proportion of false positives.

## Results

**Fig. 1:**
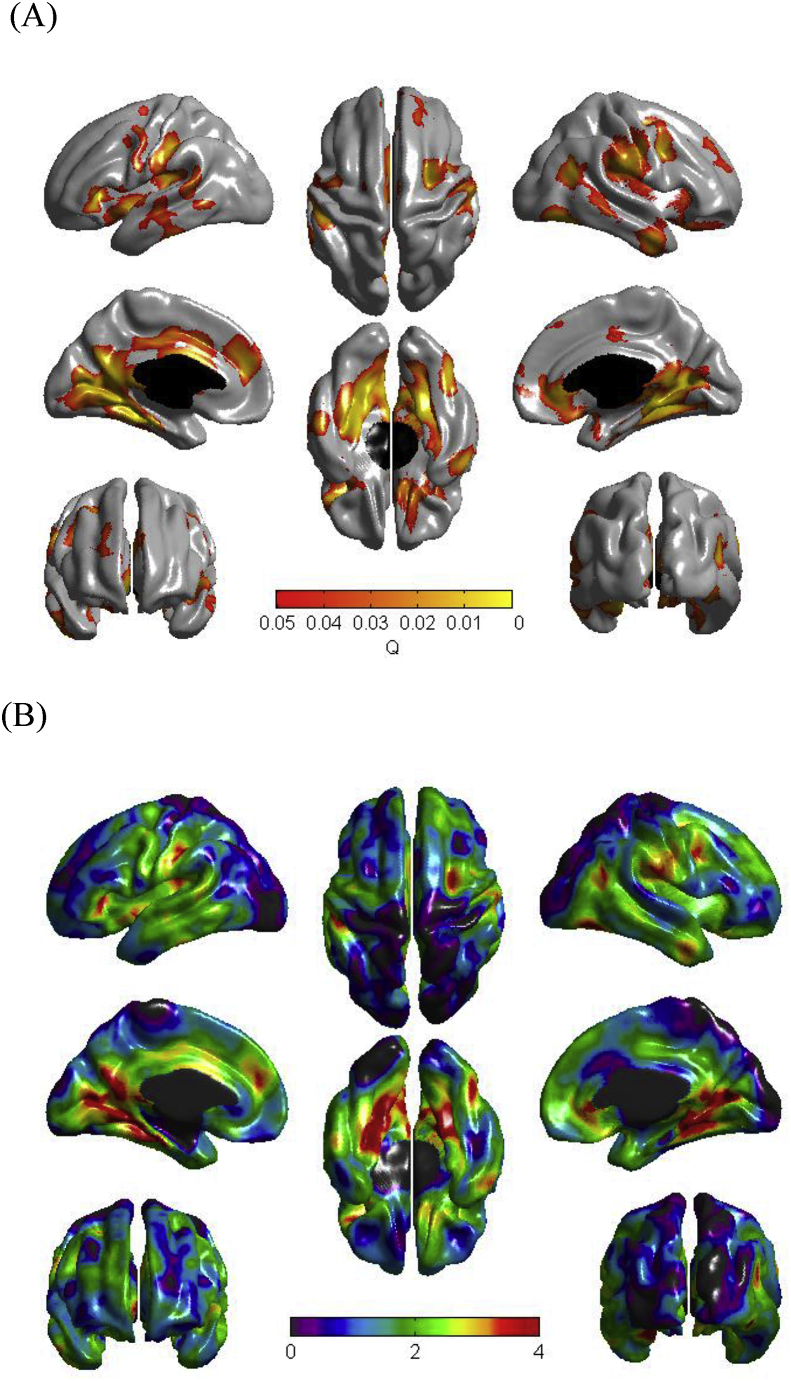
Results of cortical thickness regressed against (A) Big Five conscientiousness (Q map), (B) Big Five conscientiousness (t map). A false discovery rate threshold of 0.05 is used to control for multiple comparisons. Colors, representing Q/t values, are superimposed on an average surface template. Results are corrected for sex, age in days at brain scanning, intracranial volume, the other four Big Five traits/plasticity, and general intelligence. A Q-map illustrates areas of significance at P < 0.05 after adjusting for multiple comparisons via false discovery rate.

A widespread pattern of statistically significant associations was observed between conscientiousness and cortical thickness. The most prominent of these associations were noted with bilateral parahippocampal gyrus, bilateral fusiform gyrus, left dorsal cingulate gyrus, right ventral anterior cingulate, right medial orbitofrontal cortex, and left dorsomedial prefrontal cortex (see Fig. 1A and B). In each case greater cortical thickness in these regions related to higher levels of conscientiousness.

For the other Big Five traits and for the meta-traits stability and plasticity, no associations with cortical thickness survived FDR correction. We also observed no evidence for associations between cortical surface area and gray matter for any of the personality traits. And we also observed no evidence for sex-dependent associations between our brain measures and personality.

In a set of exploratory steps we reran our initial models but now excluding the non-target personality traits and general intelligence (although still including sex, age at scanning in days, and intracranial volume). This was done to assess whether variance shared between the personality traits and between those traits and intelligence (see Supplementary Table 2) masked personality trait links to our brain measures. Conscientiousness again showed widespread associations with cortical thickness, and stability showed a link with left fusiform gyrus, which as detailed above was also noted for conscientiousness (see Supplementary Figs. 1–2), but no other significant associations were seen (see Supplementary Figs. 3–21).

**Fig. 2:**
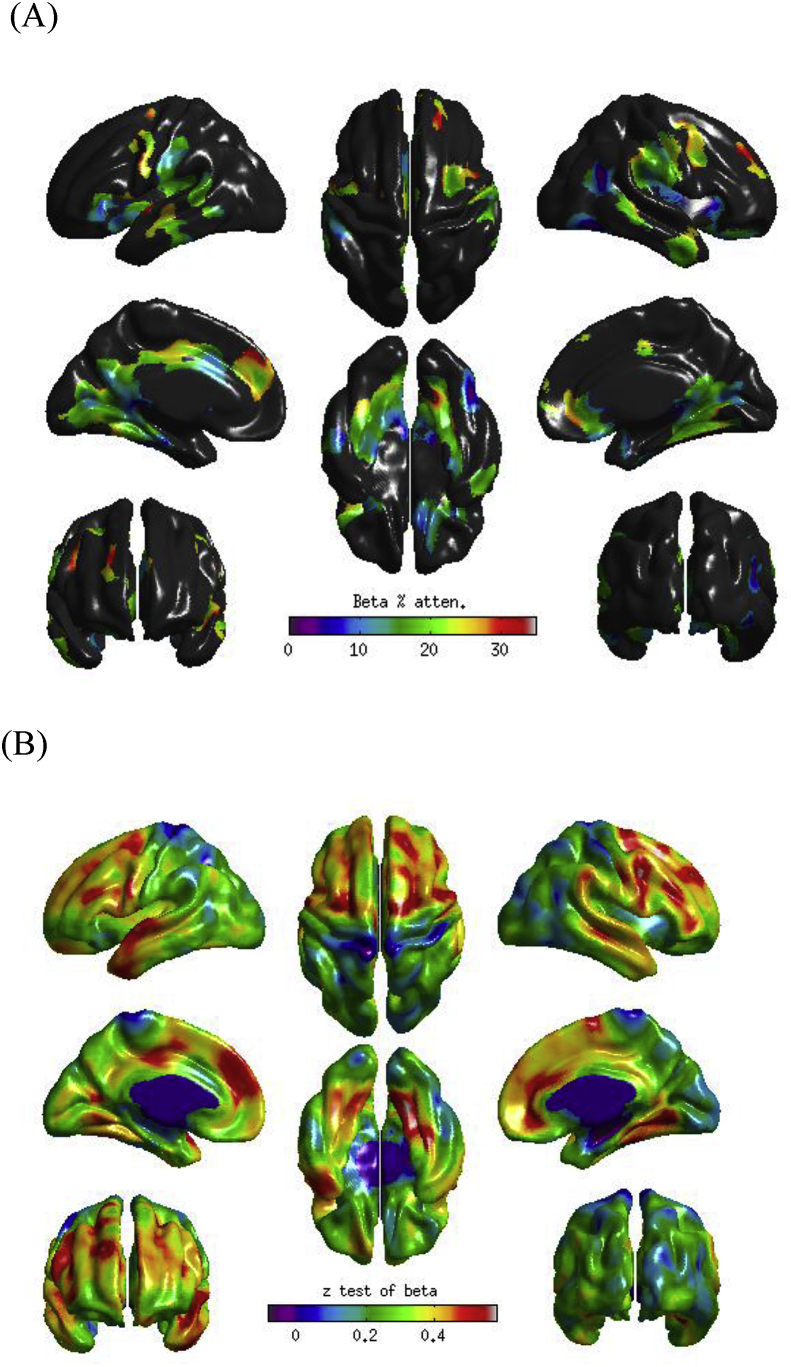
(A) Proportion of the association between Big Five conscientiousness and cortical thickness that is accounted for by the four allostatic load variables and smoking status; (B) formal test of attenuation for the association between conscientiousness and cortical thickness following the inclusion of the four allostatic load variables and smoking status.

**Fig. 3:**
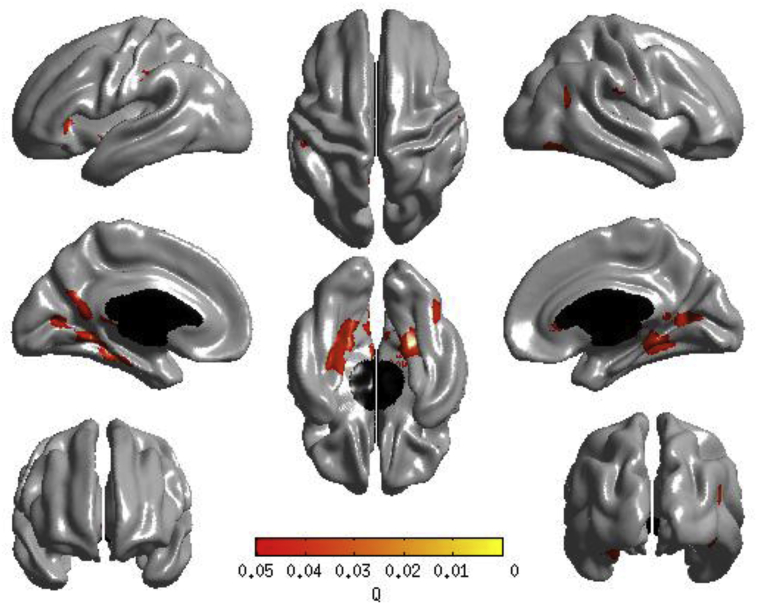
Results of cortical thickness regressed against Big Five conscientiousness. A false discovery rate threshold of 0.05 is used to control for multiple comparisons. Colors, representing Q values, are superimposed on an average surface template. Results are corrected for sex, age in days at brain scanning, intracranial volume, the other four Big Five traits, general intelligence, the four allostatic load variables, and smoking status. A Q-map illustrates areas of significance at P < 0.05 after adjusting for multiple comparisons via false discovery rate.

We next sought to examine whether the observed associations between conscientiousness and cortical thickness were accounted for by allostatic load and smoking status. To this end, we re-ran the model including the four measures of allostatic load and the participant's smoking status. The inclusion of these variables modestly-to-moderately attenuated (by up to 30%) the relationships between conscientiousness and cortical thickness (see Fig. 2A) with the majority of the frontal cortical thickness associations no longer apparent (see Fig. 3). It is noteworthy, however, that the attenuation of the association between conscientiousness and cortical thickness was not statistically significant in any region (see Fig. 2B).

## Discussion

We observed widespread positive associations between cortical thickness and conscientiousness. These associations included bilateral parahippocampal gyrus, bilateral fusiform gyrus, left dorsal cingulate gyrus, right ventral anterior cingulate, right medial orbitofrontal cortex, and left dorsomedial prefrontal cortex. We also observed a positive association between cortical thickness in left fusiform gyrus and the meta-trait stability, although only in an exploratory step when plasticity and general intelligence were excluded from the model. No other Big Five or plasticity associations were observed, nor did we observe any evidence for associations between cortical surface area or volume and any of the personality traits. And no evidence was observed for sex-dependent associations, in contrast to some recent work (Nostro et al., 2017).

Controlling for allostatic load attenuated the associations between cortical thickness and conscientiousness by up to 30% (the attenuation was greatest in frontal regions, including left medial superior frontal gyrus and right rostral/subgenual anterior cingulate). The magnitudes of these attenuations were not in and of themselves statistically significant, although the power to detect these likely much more subtle effects – i.e. capable of changing a test statistic from being just below the nominal threshold for significance to being just above – was limited in the current study.

The observed links between conscientiousness and cortical thickness provide some convergence with previous reports. In particular, our finding of a positive link with regions within left prefrontal cortex is consistent with prior work in the field (DeYoung et al., 2010; Kapogiannis et al., 2013; Riccelli et al., 2017). However, our findings also show divergence from prior work: for example, whereas a negative association between gray matter in fusiform gyrus and conscientiousness has been reported (DeYoung et al., 2010), here we found a positive association with conscientiousness in this region. This specific disparity may reflect the relatively old sample used in the current study. Given the well-noted links between conscientiousness and negative health behaviours (Friedman and Kern, 2014) it is conceivable that the associations observed here only become apparent in later life when a deleterious lifestyle gives rise to differential brain degeneration. However, Bjørnebekk et al. (2013: N = 265) – with a sample of mean age 50 years, and so relatively old too – also assessed cortical thickness and did not observe this association. In addition, whereas we found a positive association between conscientiousness and cortical thickness in superior temporal gyrus, Bjørnebekk et al. (2013) report a negative association in this region. As such, while our study has notable strengths with regard to our analytical approach and relatively large sample size, and thus provides a strong approximation of the nature of the association between cortical thickness and conscientiousness, future work is still required in order to provide more definitive conclusions.

Why are the current findings mostly specific to conscientiousness? Reflecting on the characteristic properties of the Big Five traits may provide an explanation. Specifically, conscientiousness is commonly conceived as a trait closely linked to executive control and task-oriented planning (Roberts et al., 2009). These qualities are typically associated with cortical structures (e.g. Westlye et al., 2011a) and so the widespread links observed here between conscientiousness and cortical thickness are consistent with this account. In contrast, traits such as neuroticism and extraversion might more closely reflect sub-cortical anatomy.

These cross-sectional results necessitate discussion regarding the causal direction of the observed associations. One possibility is that individual differences in brain structure give rise to trait differences in conscientiousness, which in turn increase allostatic load (e.g. via deleterious lifestyle choices). An alternative – although not incompatible – perspective might posit that conscientiousness influences allostatic load, which in turn impacts on brain health (e.g. health difficulties that emerge with bodily wear and tear might over time diminish goal-oriented behaviors that reflect conscientiousness). It is also plausible that allostatic load leads to brain degeneration, which in turn alters personality, consistent with recent longitudinal work showing that higher levels of allostatic load were related to steeper declines in conscientiousness over a 4-year period (Stephan et al., 2016). Cross-sectional data, such as that presented here, cannot parse these process models. Nonetheless, our findings, highlighting links between cortical thickness, conscientiousness, and allostatic load provide a strong basis for future research using more sophisticated approaches – e.g. longitudinal study designs – to establish causal primacy.

The current results likely have broader relevance. For example, recent meta-analytic work has reported that individuals in the lowest quartile of conscientiousness showed a threefold increase in Alzheimer's disease (Terracciano et al., 2014). Moreover, there is a well-noted link between the parahippocampal region and Alzheimer's disease (De Leon et al., 2004), which mirror some of the brain associations observed here for conscientiousness. As noted above, causal pathways cannot be inferred in our cross-sectional data – however, the current findings suggest a nexus between conscientiousness, Alzheimer's disease, and parahippocampal cortex.

Specific limitations require mention. Firstly, our sample consisted of older adults and this may constrain the generalizability of these findings. Secondly, other neural phenotypes might provide for additional prediction. These phenotypes would include cortical folding (which is not yet available at the local level via CIVET) and white matter microstructure, which have both shown links to personality in the few such studies reported to date (e.g. Lewis et al., 2016; McIntosh et al., 2013; Riccelli et al., 2017; Westlye et al., 2011b).

In summary, we examined links between brain gray matter and human personality trait differences, as assessed via the Big Five framework. We found evidence for positive links between conscientiousness and cortical thickness in a range of brain areas, including bilateral parahippocampal gyrus, bilateral fusiform gyrus, left dorsal cingulate gyrus, right ventral anterior cingulate, right medial orbitofrontal cortex, and left dorsomedial prefrontal cortex. These associations were modestly-to-moderately attenuated when controlling for allostatic load and smoking status, although the magnitudes of these attenuations were modest and not in and of themselves statistically significant. We also observed suggestive evidence for an association between left fusiform gyrus and the meta-trait stability. We did not observe any neuroanatomical links for the other Big Five traits or for the meta-trait plasticity. In addition, sex was not a moderator of the association between brain cortical thickness and personality. Collectively, these findings provide important insights concerning the neural correlates of personality traits and indicate that allostatic load/smoking, cortical thickness, and conscientiousness share important links. Future research is now recommended to build upon these results in order to establish the causal nature of the relationship between these variables.

## Appendix A Supplementary data

Supp materials Supp materials
