# Supplementary material for: Widespread associations between trait conscientiousness and thickness of brain cortical regions
Source: Neuroimage. 2018 Aug 1;176:22–8. doi: 10.1016/j.neuroimage.2018.04.033 (PMC5986708; doi:10.1016/j.neuroimage.2018.04.033)
Supplement: Supp materials [file mmc1.docx]

Supplementary Table 1.Overview of regional gray matter volume, cortical thickness, and surface area correlates of Big Five personality traits.

| **Authors** | **Agreeableness** | **Conscientiousness** | **Extraversion** | **Neuroticism** | **Openness** | **Sample** |
| --- | --- | --- | --- | --- | --- | --- |
| Bjørnebekk et al (2013) | None | None | ***CT***: L inferior frontal gyrus (-) | None | None | N=265; Norway; Mean age=50yrs |
| Coutinho et al (2013) | ***GMV***: R inferior parietal gyrus (-); L middle occipital gyrus (-); L posterior cingulate gyrus (-) | No test | ***GMV***: L+R middle frontal (-); L+R orbitofrontal gyri (-); R inferior frontal gyrus (-); R superior frontal gyrus (-) | No test | No test | N=52; Portugal; Mean age=25yrs |
| Cremers et al (2011) | No test | No test | ***GMV***: R medial orbitofrontal cortex (+); R amygdala (+)§ | None§ | No test | N=65; The Netherlands; Mean age=41yrs |
| DeYoung et al (2010) | ***GMV***: L posterior cingulate (+); R fusiform gyrus (+); L superior temporal sulcus (-) | **GMV**: L middle frontal gyrus (+); R fusiform gyrus (-) | ***GMV***: R medial orbitofrontal cortex (+) | ***GMV***: L medial temporal lobe (-); R medial frontal gyrus (-); R midcingulate gyrus (+); L middle temporal gyrus (+); R precentral gyrus (-); R cerebellum (+) | None | N=116; USA; Mean age=23yrs |
| Holmes et al (2013) | No Test | No Test | No Test | ***GMV***: L+R amygdala (+)  ***CT***: L medial prefrontal cortex (-) | No Test | N=1050; USA; Mean age=21yrs |
| Kapogiannis et al (2013) | ***GMV***: R orbitofrontal cortex (+); R middle temporal pole (+); L superior parietal cortex (+); L+R superior medial frontal gyrus (-); L middle frontal gyrus (-); L parahippocampal gyrus (-); R calcarine gyrus (-); L superior temporal gyrus (-) | **GMV**: L+R dorsomedial prefrontal cortex/superior frontal gyrus (+); R postcentral gyrus (+); L precuneus (+); R inferior frontal gyrus (+); R superior temporal gyrus (+); R precentral gyrus (+); L superior frontal gyrus (+); L anterior cingulate gyrus (+); L caudate nucleus (+); L lingual gyrus (+); L hippocampus (+); R middle temporal pole (-); L superior temporal gyrus (-); L+R frontal pole (-); L superior parietal lobule (-); L calcarine gyrus (-); R postcentral gyrus (-); R medial orbitofrontal cortex (-) | ***GMV***: L temporal cortex (+); L dorsolateral prefrontal cortex (+); L anterior cingulate cortex (+); L supplemental motor area (+); L superior temporal gyrus (+); R insular cortex (+); L parahippocampal cortex (-); L inferior occipital cortex (-); L superior parietal lobule (-) | ***GMV***: R lingual cortex (+); R fusiform cortex (+); R middle occipital cortex (+); R precentral cortex (+); R orbitofrontal cortex (-); R rolandic opperculum cortex (-); R middle frontal gyrus (-); R parahippocampal cortex (-); R middle temporal cortex (-) | ***GMV***: R fronto-polar cortex (+); L thalamus (+); L+R fusiform gyrus (-); L fronto-insular cortex (-); R superior frontal cortex (-); L supplemental motor area (-); L post-central cortex (-); R precuneus (-); L inferior parietal cortex (-) | N=87; USA; Mean age=72yrs |
| Lewis et al (2014) | No test | No test | ***GMV***: L+R amygdala (+)§ | None§ | No test | N=486; USA; Mean age=55yrs |
| Liu et al (2013) | None | None | None | None | None | N=227; Germany; Mean age=26yrs |
| Lu et al (2014) | No Test | No test | ***GMV***: L+R amygdala (-); L+R parahippocampal gyrus (-); R middle temporal gyrus (-); L superior frontal gyrus (-) | ***GMV***: R cerebellum (+); L superior frontal gyrus (-) | No test | N=71; China; Mean age=21yrs |
| Nostro et al (2017) | None | None* | None* | None* | None | N=364; USA; Mean age=29yrs |
| Riccelli et al (2017) | ***GMV***: L rostral middle frontal cortex (-)  ***CT***: L caudal middle frontal (-); L pars opercularis (-); L superior frontal (-); R rostral middle frontal (-)  ***SA***: R fusiform (-) | ***GMV***: L lateral occipital (-); R inferior temporal (-)  ***CT***: L+R middle frontal cortex (+); R precuneus (+)  ***SA***: L lateral occipital (-); R middle temporal (-) | ***GMV***: L superior temporal gyrus (-); R entorhinal cortex (-)  ***CT***: L precuneus (+)  ***SA***: R superior temporal (-) | ***GMV***: L middle temporal cortex (-); L superior temporal cortex (-); L lateral occipital cortex (-); R fusiform gyrus (-)  ***CT***: L supra-marginal gyrus (+); L+R superior prefrontal cortex (+); R superior parietal cortex (+); R postcentral cortex (-); R superior temporal cortex (+)  ***SA***: L+R middle temporal (-); L rostral middle frontal (-); L+R superior frontal (-); L cuneus (-); R superior parietal (-); R frontal pole (-) | ***GMV***: L+R inferior temporal cortex (+); L temporal pole (+)  ***CT***: L+R rostral middle frontal cortex (-); L lateral occipital gyrus (-); L rostral anterior cingulate cortex (-); R postcentral cortex (-); R inferior parietal cortex (-); R superior frontal cortex (-)  ***SA***: L inferior temporal (+); R postcentral (+); R lateral occipital (+); R inferior parietal (+) | N=507; USA; Mean age=29yrs |

Note. GMV = gray matter volum; CT = cortical thickness; SA = surface area; (-) = negative association; (+) = positive association; No test = no test was performed for this trait; None = no significant associations between cortical thickness, surface area, or gray matter volume for this trait; * significant sex differences noted (refer to paper for full results); § = this test was restricted to predetermined regions of interest and not whole-brain search (refer to paper for full details).

Supplementary Table 2. Descriptive statistics of study variables.

|  | Mean | Range | SD |
| --- | --- | --- | --- |
| Agreeableness | 30.73 | 30 | 5.65 |
| Conscientiousness | 27.49 | 35 | 6.16 |
| Extraversion | 21.61 | 39 | 6.88 |
| Emotional stability | 25.26 | 38 | 7.51 |
| Intellect | 23.82 | 35 | 5.88 |
| Stability | 27.83 | 25 | 4.52 |
| Plasticity | 22.71 | 28 | 5.34 |

Note. SD = standard deviation; general intelligence and the allostatic load variables were operationalized as a principle component and factor scores, respectively, thus means were approximately zero in all cases.

Supplementary Table 3. Correlations between Big Five traits and allostatic load variables.

|  | A | C | Es | Ex | I | plas | stab | GI |
| --- | --- | --- | --- | --- | --- | --- | --- | --- |
| C | .26* |  |  |  |  |  |  |  |
| Es | .17* | .27* |  |  |  |  |  |  |
| Ex | .33* | .13* | .22* |  |  |  |  |  |
| I | .30* | .14* | .12* | .40* |  |  |  |  |
| plas | .37* | .16* | .21* | .86* | .81* |  |  |  |
| stab | .63* | .71* | .75* | .31* | .26* | .34* |  |  |
| GI | .05 | .07 | .19* | .06 | .26* | .18* | .15* |  |
| ALg | -.07 | -.11* | -.05 | .03 | .06 | .05 | -.11* | -.11* |
| ALinf | -.02 | -.09* | .00 | -.04 | -.07 | -.06 | -.05 | -.09* |
| ALmet | -.11* | -.05 | .00 | -.02 | -.04 | -.04 | -.07 | .01 |
| ALbp | .03 | .05 | .06 | -.06 | -.01 | -.04 | .07 | .02 |
| Smoke | -.09* | -.08 | -.04 | .02 | -.02 | .01 | -.09* | -.09* |

Note. Correlations between allostatic load variables are derived from bifactor scores (see main text) and so are uncorrelated and thus omitted here; A = agreeableness; C = conscientiousness; Es = emotional stability; Ex = extraversion; I = intellect; plas = plasticity; stab = stability; AL = allostatic load; GI = general intelligence; g = general factor; inf = inflammation; met = metabolic; bp = blood pressure; smoke = smoking status (Never=1; Ever = 2); * = p < .01.

Supplementary Figure 1. Results of cortical thickness regressed against (A) Big Five conscientiousness (Q map), (B) Big Five conscientiousness (t map). A false discovery rate threshold of 0.05 is used to control for multiple comparisons. Colors, representing Q/t values, are superimposed on an average surface template. Results are corrected for sex, age in days at brain scanning, and intracranial volume.

(A)


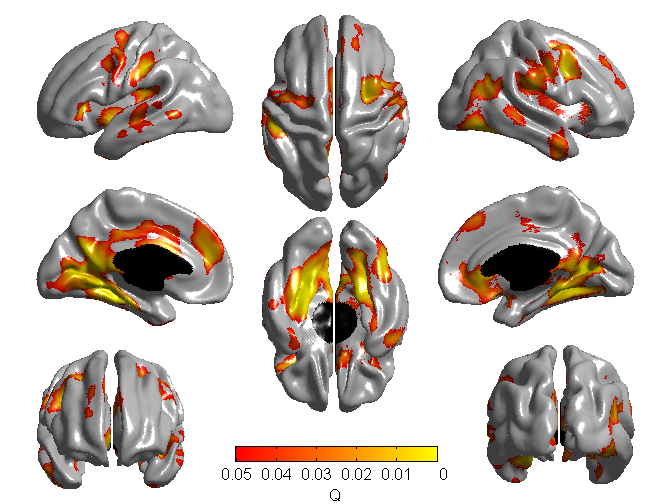


(B)


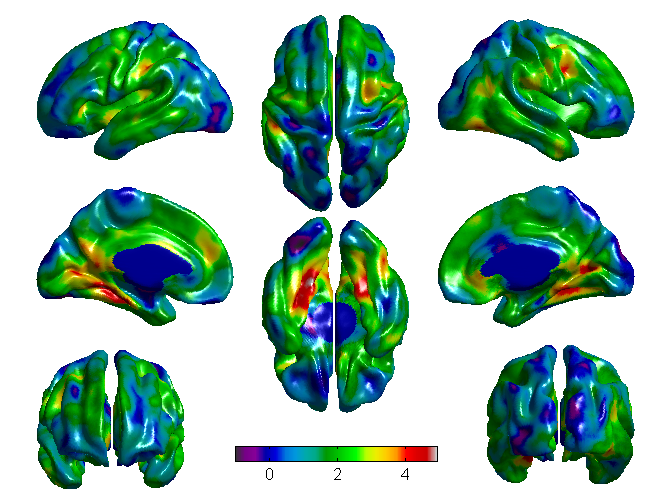


Supplementary Figure 2. Results of cortical thickness regressed against (A) meta-trait stability (Q map), (B) meta-trait stability (t map). A false discovery rate threshold of 0.05 is used to control for multiple comparisons. Colors, representing Q/t values, are superimposed on an average surface template. Results are corrected for sex, age in days at brain scanning, and intracranial volume.

(A)


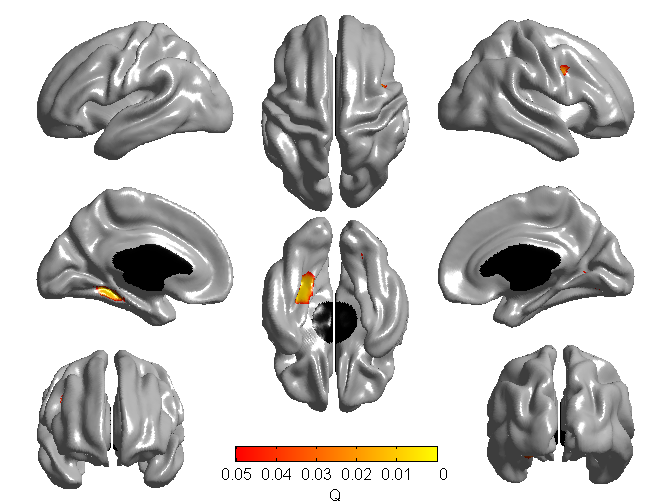


(B)


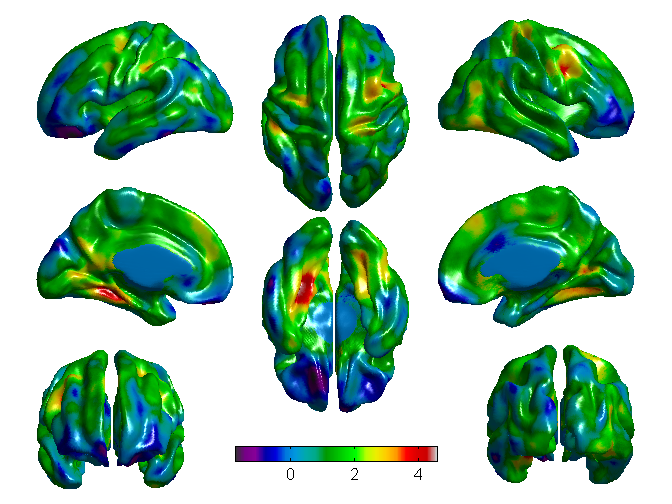


Supplementary Figure 3. Results of cortical thickness regressed against Big Five agreeableness (t map). A false discovery rate threshold of 0.05 is used to control for multiple comparisons. Colors, representing t values, are superimposed on an average surface template. Results are corrected for sex, age in days at brain scanning, and intracranial volume. No significant associations were observed (and thus we do not present a Q map here).


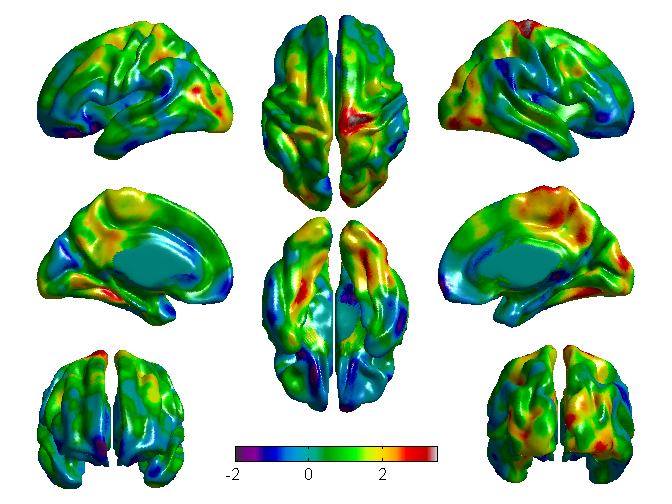


Supplementary Figure 4. Results of cortical thickness regressed against Five emotional stability (t map). A false discovery rate threshold of 0.05 is used to control for multiple comparisons. Colors, representing t values, are superimposed on an average surface template. Results are corrected for sex, age in days at brain scanning, and intracranial volume. No significant associations were observed (and thus we do not present a Q map here).


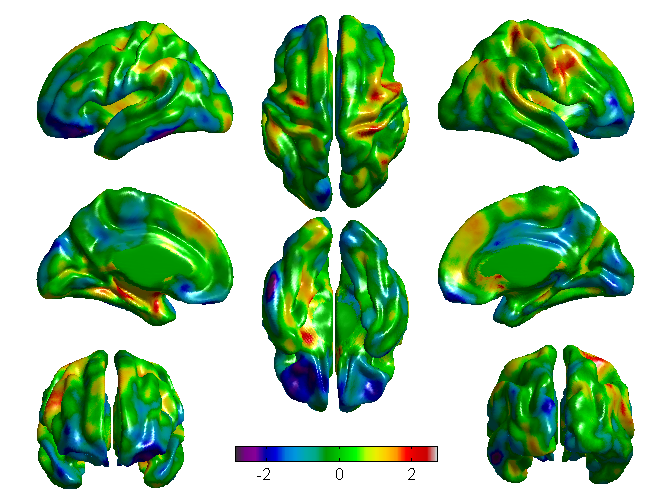


Supplementary Figure 5. Results of cortical thickness regressed against Big Five extraversion (t map). A false discovery rate threshold of 0.05 is used to control for multiple comparisons. Colors, representing t values, are superimposed on an average surface template. Results are corrected for sex, age in days at brain scanning, and intracranial volume. No significant associations were observed (and thus we do not present a Q map here).


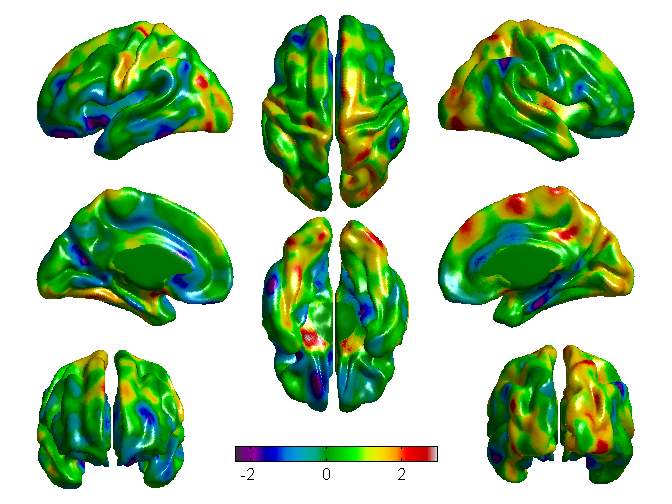


Supplementary Figure 6. Results of cortical thickness regressed against Big Five intellect/openness (t map). A false discovery rate threshold of 0.05 is used to control for multiple comparisons. Colors, representing t values, are superimposed on an average surface template. Results are corrected for sex, age in days at brain scanning, and intracranial volume. No significant associations were observed (and thus we do not present a Q map here).


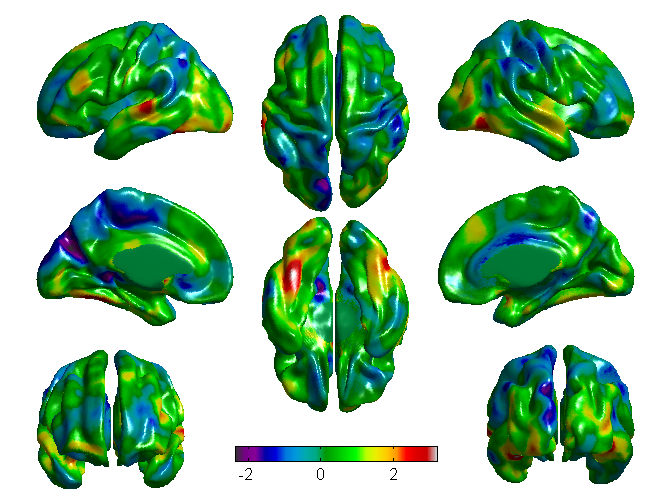


Supplementary Figure 7. Results of cortical thickness regressed against meta-trait plasticity (t map). A false discovery rate threshold of 0.05 is used to control for multiple comparisons. Colors, representing t values, are superimposed on an average surface template. Results are corrected for sex, age in days at brain scanning, and intracranial volume. No significant associations were observed (and thus we do not present a Q map here).


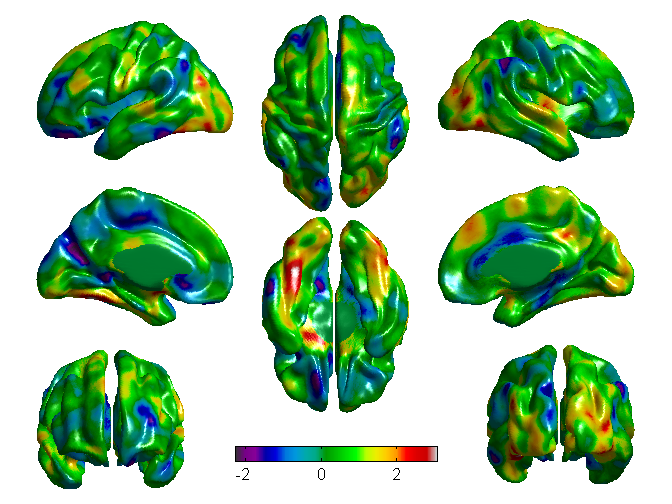


Supplementary Figure 8. Results of surface area regressed against Big Five agreeableness (t map). A false discovery rate threshold of 0.05 is used to control for multiple comparisons. Colors, representing t values, are superimposed on an average surface template. Results are corrected for sex, age in days at brain scanning, and intracranial volume. No significant associations were observed (and thus we do not present a Q map here).


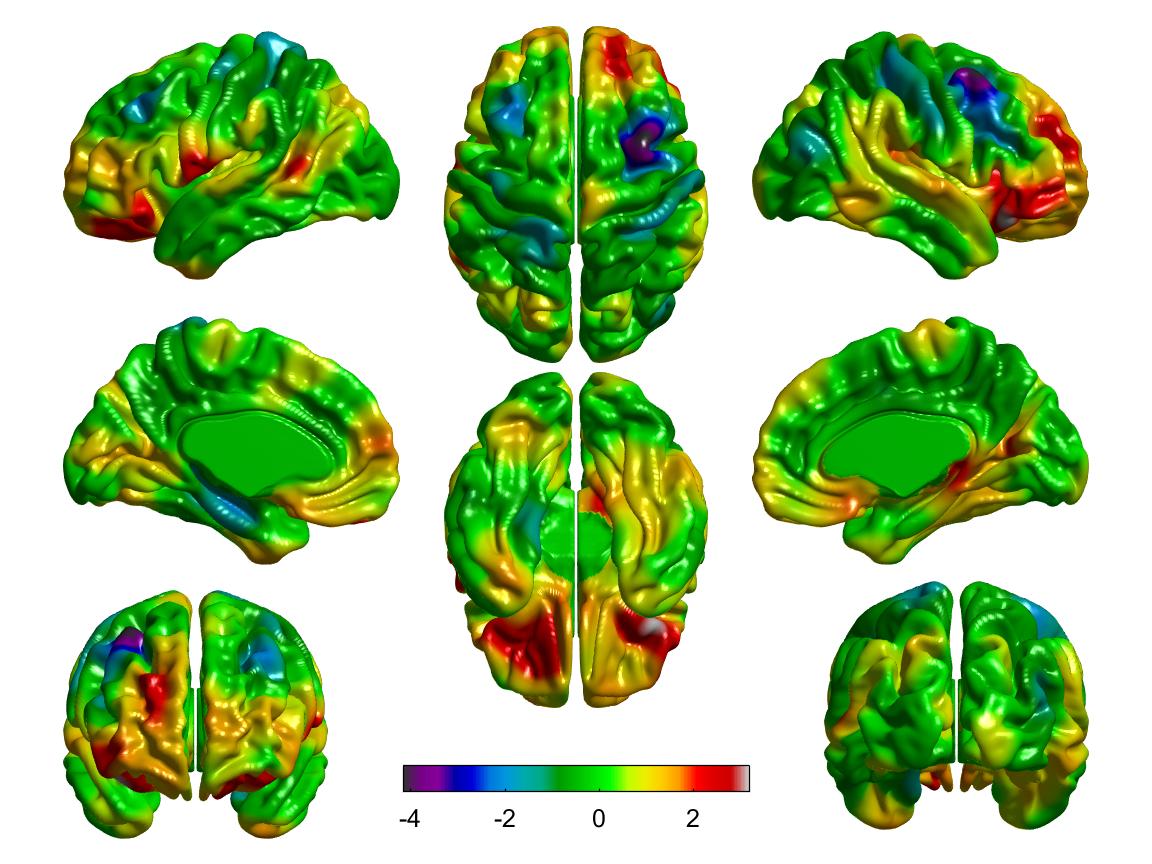


Supplementary Figure 9. Results of surface area regressed against Big Five conscientiousness (t map). A false discovery rate threshold of 0.05 is used to control for multiple comparisons. Colors, representing t values, are superimposed on an average surface template. Results are corrected for sex, age in days at brain scanning, and intracranial volume. No significant associations were observed (and thus we do not present a Q map here).


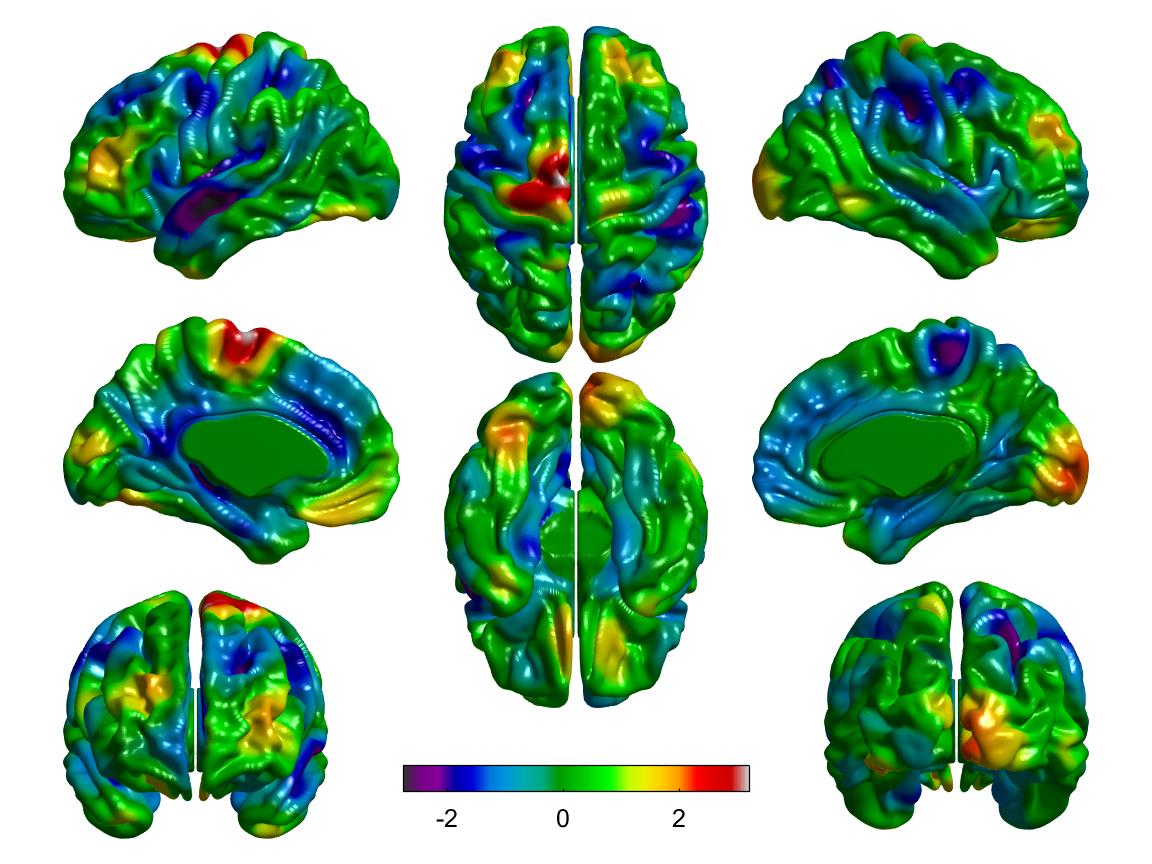


Supplementary Figure 10. Results of surface area regressed against Big Five emotional stability (t map). A false discovery rate threshold of 0.05 is used to control for multiple comparisons. Colors, representing t values, are superimposed on an average surface template. Results are corrected for sex, age in days at brain scanning, and intracranial volume. No significant associations were observed (and thus we do not present a Q map here).


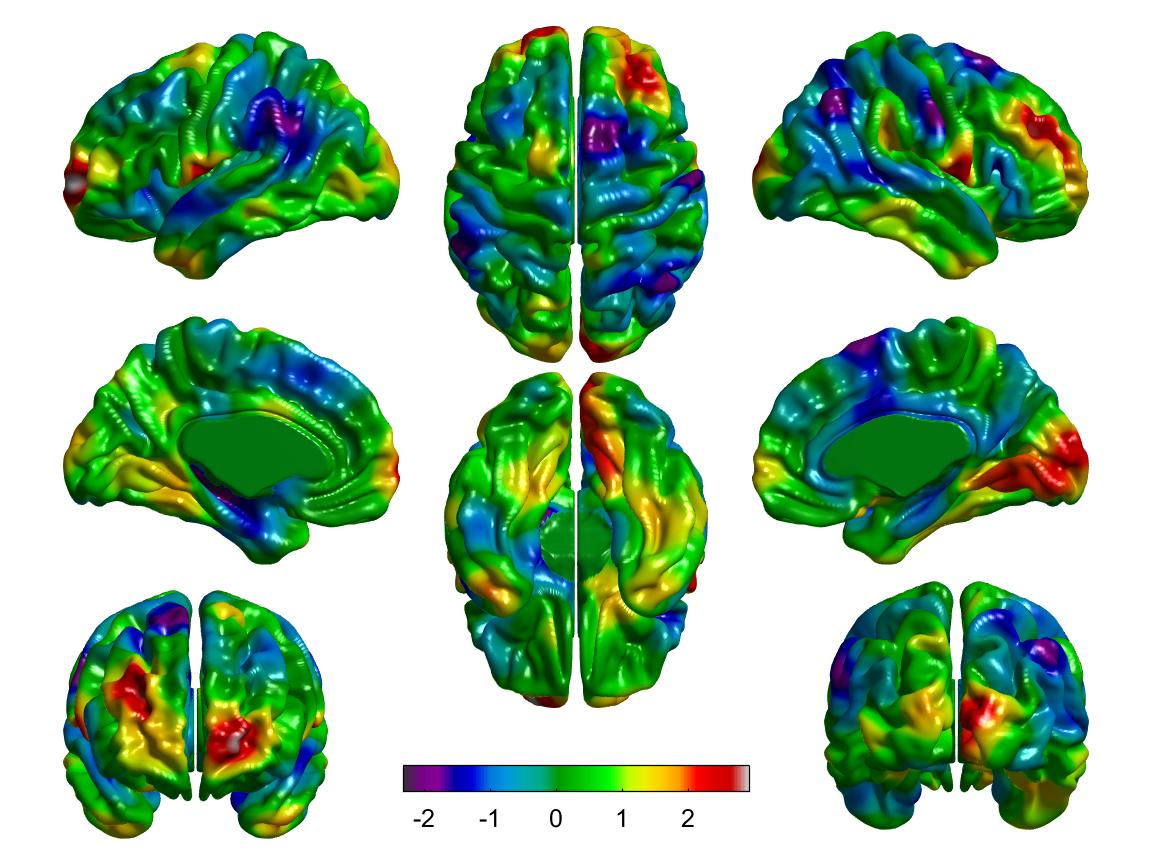


Supplementary Figure 11. Results of surface area regressed against Big Five extraversion (t map). A false discovery rate threshold of 0.05 is used to control for multiple comparisons. Colors, representing t values, are superimposed on an average surface template. Results are corrected for sex, age in days at brain scanning, and intracranial volume. No significant associations were observed (and thus we do not present a Q map here).


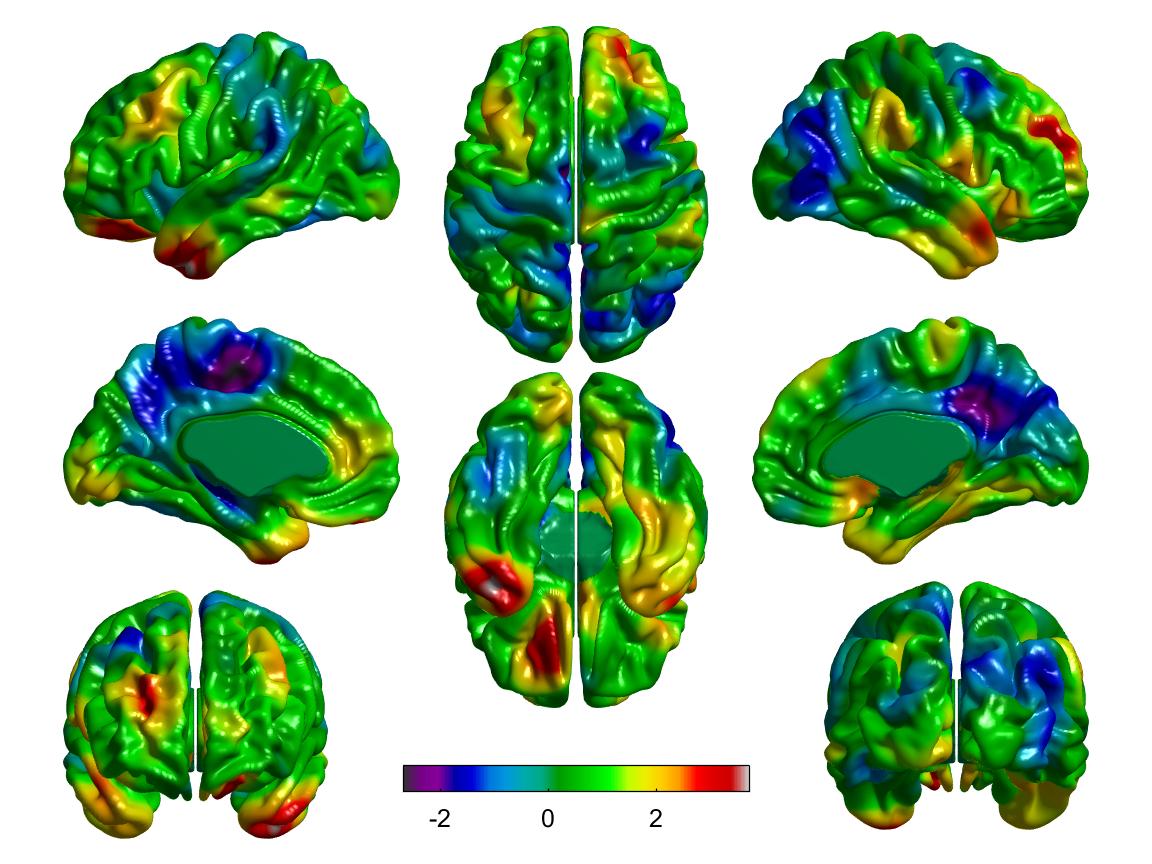


Supplementary Figure 12. Results of surface area regressed against Big Five intellect/stability (t map). A false discovery rate threshold of 0.05 is used to control for multiple comparisons. Colors, representing t values, are superimposed on an average surface template. Results are corrected for sex, age in days at brain scanning, and intracranial volume. No significant associations were observed (and thus we do not present a Q map here).


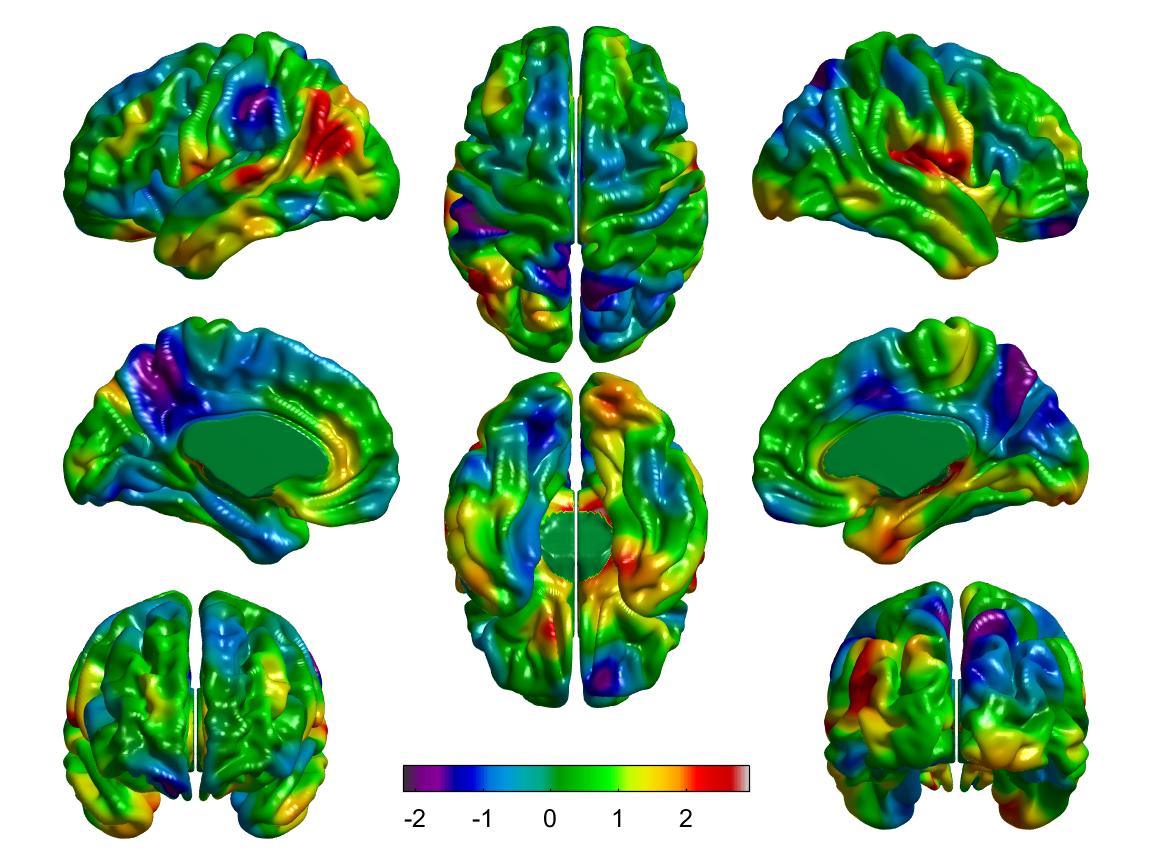


Supplementary Figure 13. Results of surface area regressed against meta-trait plasticity (t map). A false discovery rate threshold of 0.05 is used to control for multiple comparisons. Colors, representing t values, are superimposed on an average surface template. Results are corrected for sex, age in days at brain scanning, and intracranial volume. No significant associations were observed (and thus we do not present a Q map here).


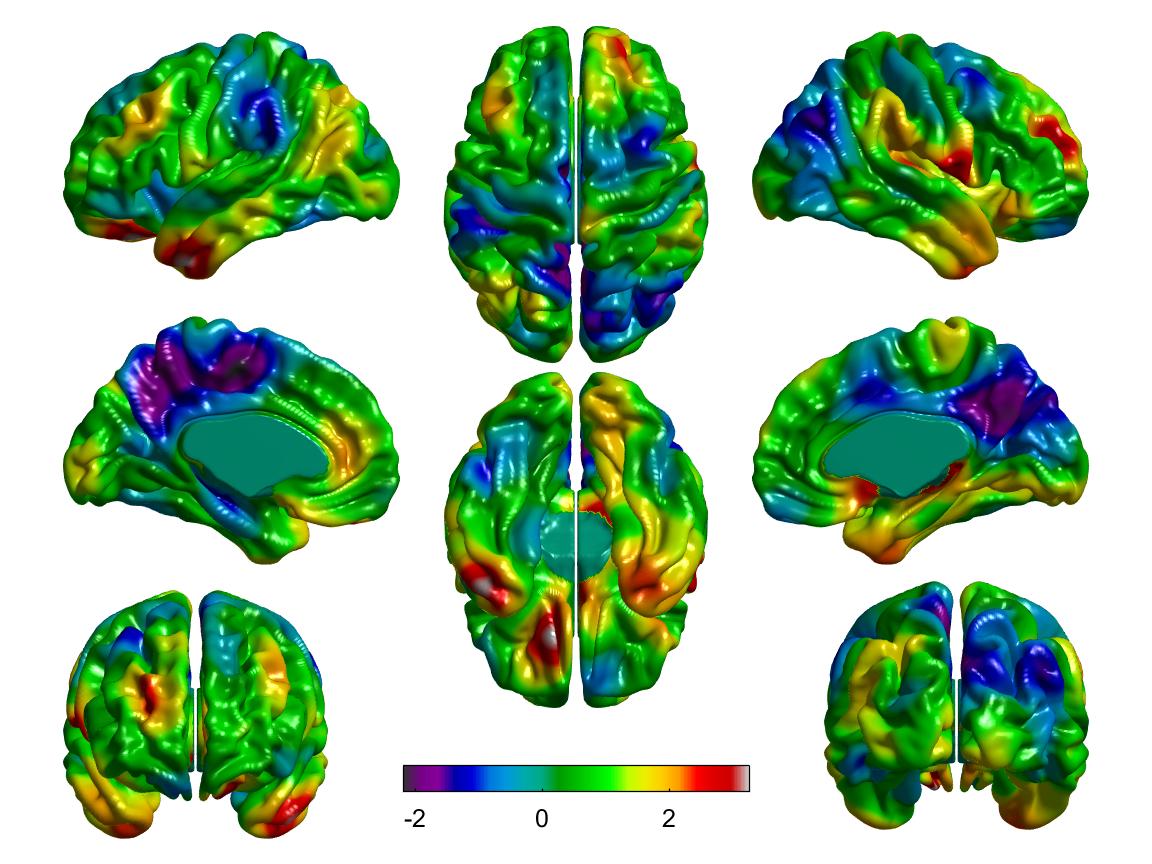


Supplementary Figure 14. Results of surface area regressed against meta-trait stability (t map). A false discovery rate threshold of 0.05 is used to control for multiple comparisons. Colors, representing t values, are superimposed on an average surface template. Results are corrected for sex, age in days at brain scanning, and intracranial volume. No significant associations were observed (and thus we do not present a Q map here).


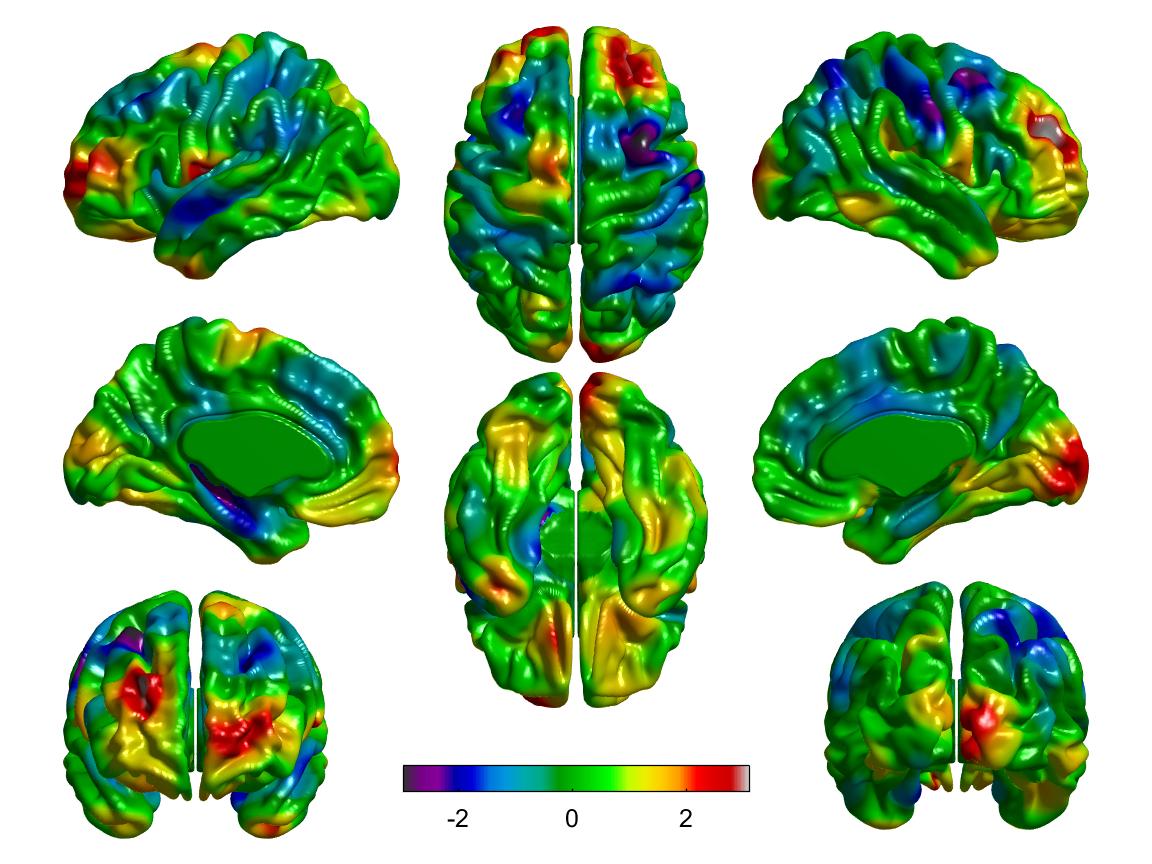


Supplementary Figure 15. Results of cortical volume regressed against Big Five agreeableness (t map). A false discovery rate threshold of 0.05 is used to control for multiple comparisons. Colors, representing t values, are superimposed on an average surface template. Results are corrected for sex, age in days at brain scanning, and intracranial volume. No significant associations were observed (and thus we do not present a Q map here).


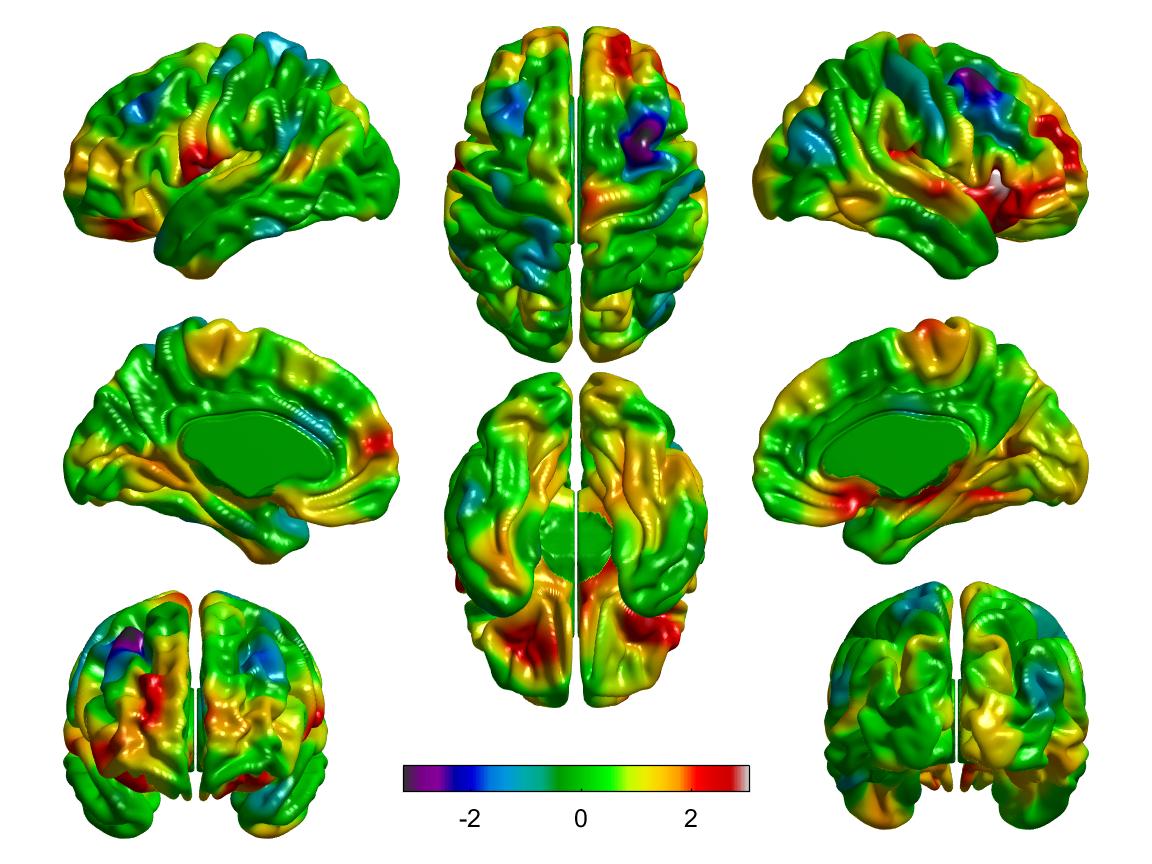


Supplementary Figure 16. Results of cortical volume regressed against Big Five conscientiousness (t map). A false discovery rate threshold of 0.05 is used to control for multiple comparisons. Colors, representing t values, are superimposed on an average surface template. Results are corrected for sex, age in days at brain scanning, and intracranial volume. No significant associations were observed (and thus we do not present a Q map here).


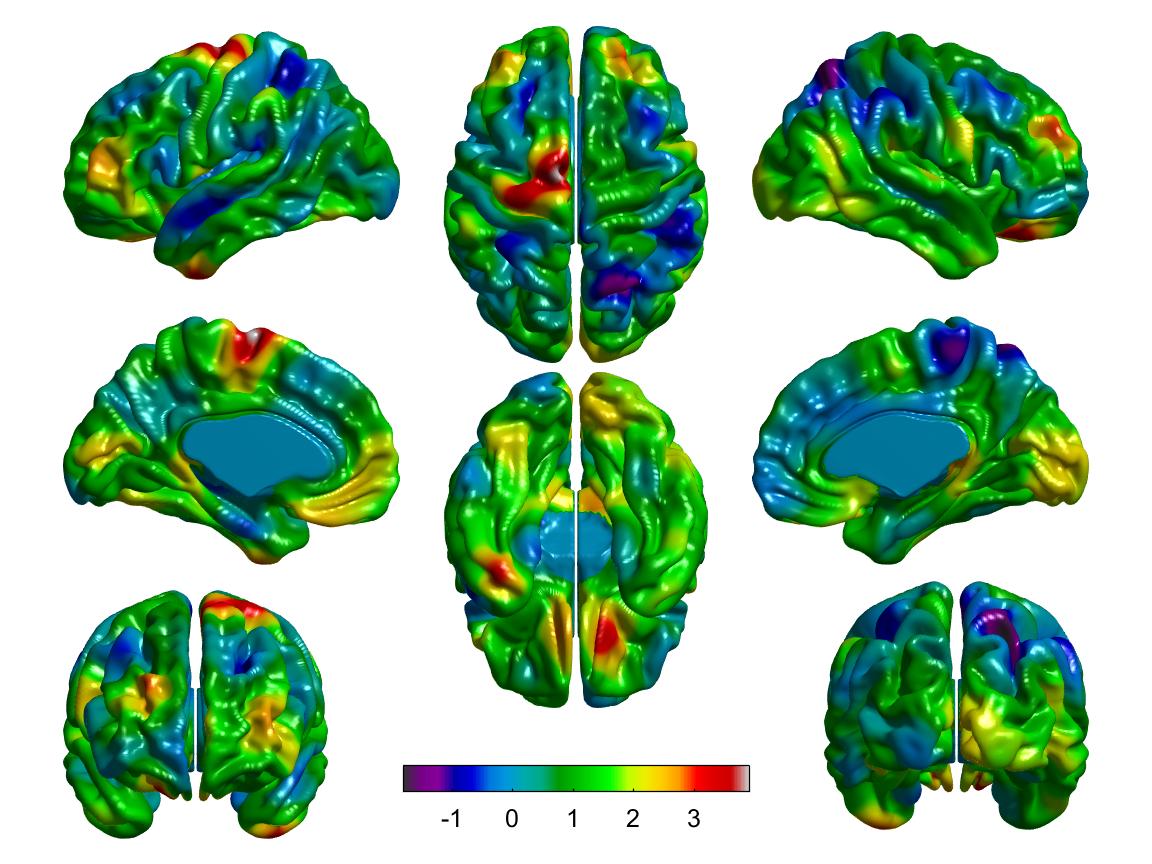


Supplementary Figure 17. Results of cortical volume regressed against Big Five emotional stability (t map). A false discovery rate threshold of 0.05 is used to control for multiple comparisons. Colors, representing t values, are superimposed on an average surface template. Results are corrected for sex, age in days at brain scanning, and intracranial volume. No significant associations were observed (and thus we do not present a Q map here).


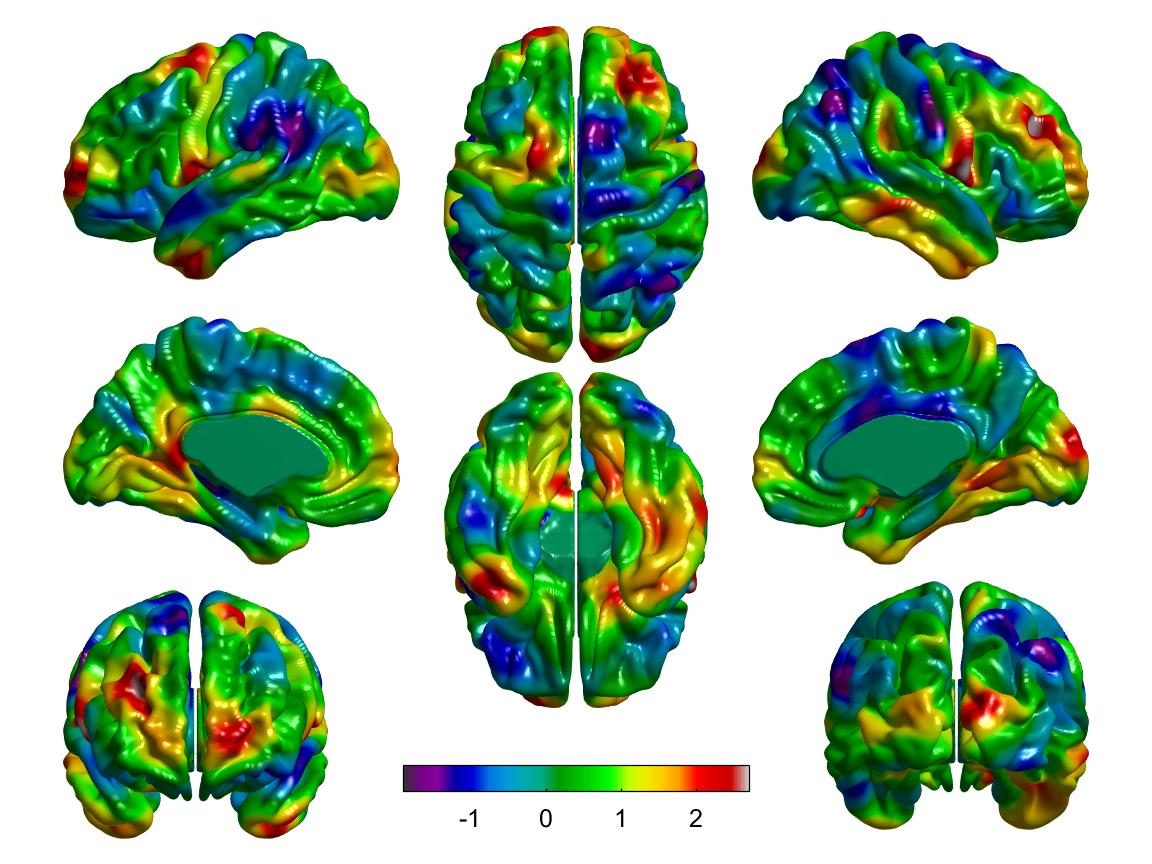


Supplementary Figure 18. Results of cortical volume regressed against Big Five extraversion (t map). A false discovery rate threshold of 0.05 is used to control for multiple comparisons. Colors, representing t values, are superimposed on an average surface template. Results are corrected for sex, age in days at brain scanning, and intracranial volume. No significant associations were observed (and thus we do not present a Q map here).


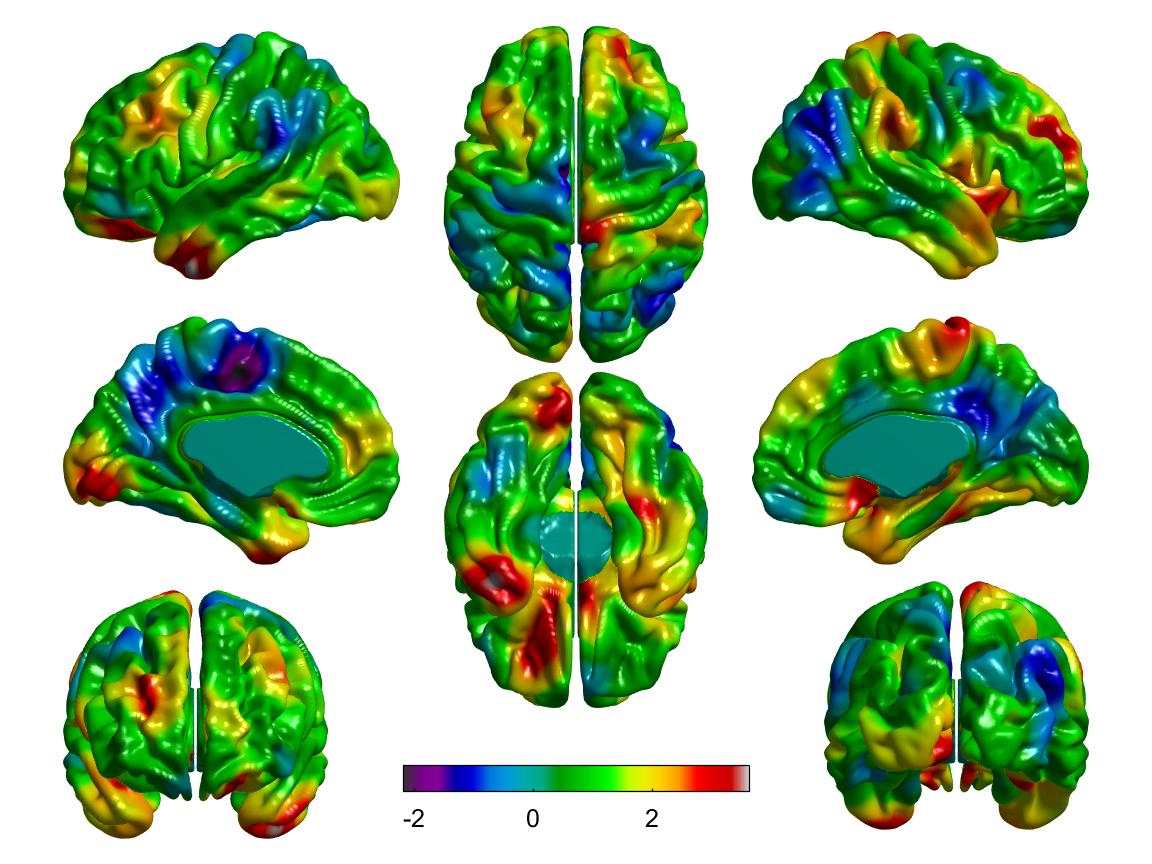


Supplementary Figure 19. Results of cortical volume regressed against Big Five intellect/stability (t map). A false discovery rate threshold of 0.05 is used to control for multiple comparisons. Colors, representing t values, are superimposed on an average surface template. Results are corrected for sex, age in days at brain scanning, and intracranial volume. No significant associations were observed (and thus we do not present a Q map here).


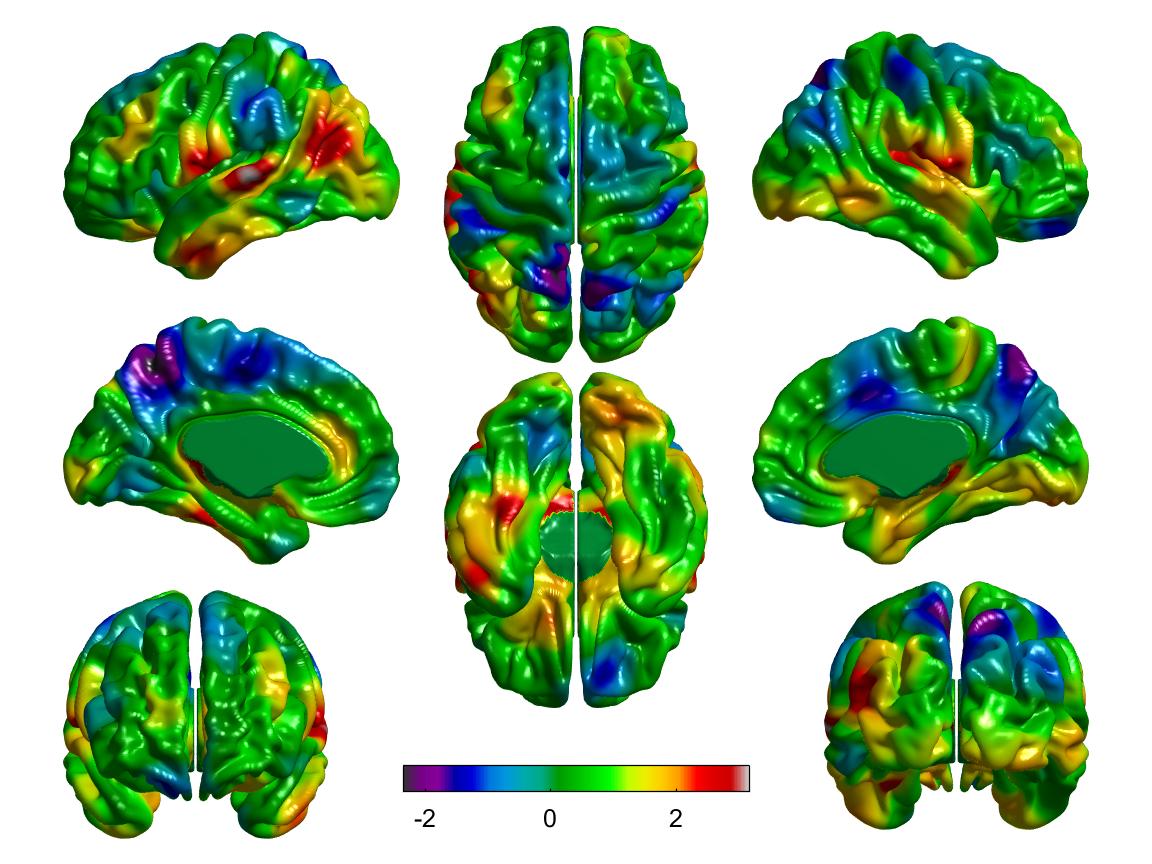


Supplementary Figure 20. Results of cortical volume regressed against meta-trait plasticity (t map). A false discovery rate threshold of 0.05 is used to control for multiple comparisons. Colors, representing t values, are superimposed on an average surface template. Results are corrected for sex, age in days at brain scanning, and intracranial volume. No significant associations were observed (and thus we do not present a Q map here).


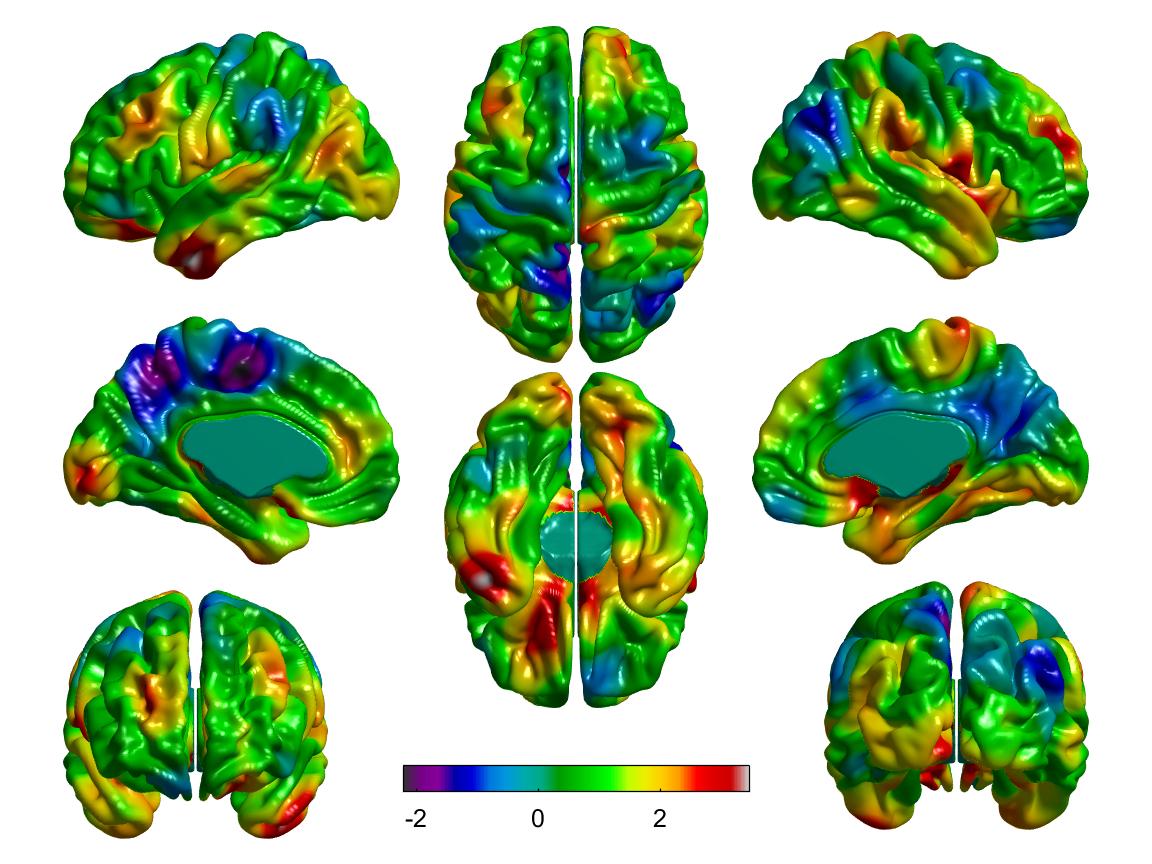


Supplementary Figure 21. Results of cortical volume regressed against meta-trait stability (t map). A false discovery rate threshold of 0.05 is used to control for multiple comparisons. Colors, representing t values, are superimposed on an average surface template. Results are corrected for sex, age in days at brain scanning, and intracranial volume. No significant associations were observed (and thus we do not present a Q map here).


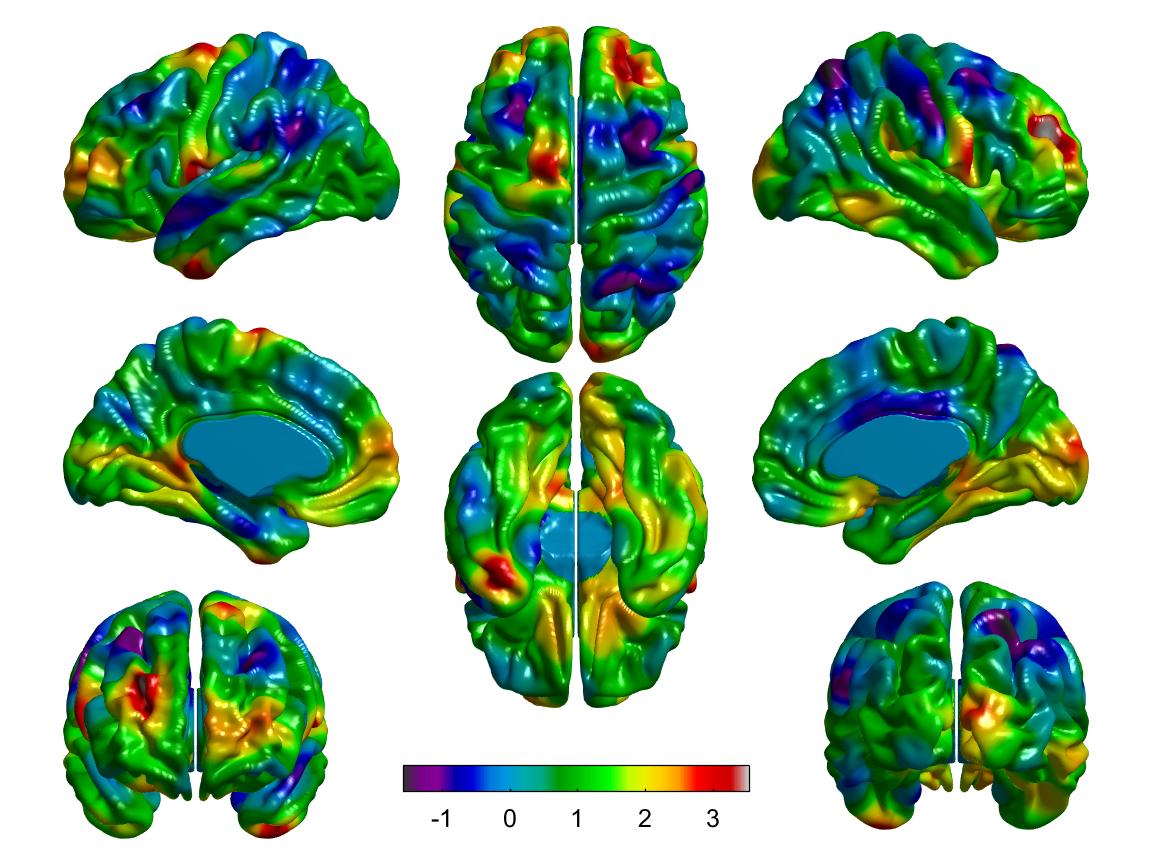


**References**

Ansell, E.B., Rando, K., Tuit, K., Guarnaccia, J., & Sinha, R. (2012). Cumulative adversity and smaller gray matter volume in medial prefrontal, anterior cingulate, and insula regions. *Biological Psychiatry, 72*, 57-64.

Arnsten, A.F. (2009). Stress signalling pathways that impair prefrontal cortex structure and function. *Nature Reviews Neuroscience, 10*, 410-422.

Bjørnebekk, A., Fjell, A. M., Walhovd, K. B., Grydeland, H., Torgersen, S., & Westlye, L. T. (2013). Neuronal correlates of the five factor model (FFM) of human personality: Multimodal imaging in a large healthy sample. *Neuroimage*, *65*, 194-208.

Cremers, H., van Tol, M. J., Roelofs, K., Aleman, A., Zitman, F. G., van Buchem, M. A., ... & van der Wee, N. J. (2011). Extraversion is linked to volume of the orbitofrontal cortex and amygdala. *PloS One, 6*, e28421.

Coutinho, J. F., Sampaio, A., Ferreira, M., Soares, J. M., & Gonçalves, O. F. (2013). Brain correlates of pro-social personality traits: a voxel-based morphometry study. *Brain Imaging and Behavior, 7*, 293-299.

DeYoung, C. G., Hirsh, J. B., Shane, M. S., Papademetris, X., Rajeevan, N., & Gray, J. R. (2010). Testing predictions from personality neuroscience brain structure and the big five. *Psychological Science, 21*, 820-828.

Holmes, A. J., Lee, P. H., Hollinshead, M. O., Bakst, L., Roffman, J. L., Smoller, J. W., & Buckner, R. L. (2012). Individual differences in amygdala-medial prefrontal anatomy link negative affect, impaired social functioning, and polygenic depression risk. *Journal of Neuroscience, 32*, 18087-18100.

Kapogiannis, D., Sutin, A., Davatzikos, C., Costa, P., & Resnick, S. (2013). The five factors of personality and regional cortical variability in the Baltimore longitudinal study of aging. *Human Brain Mapping*, *34*, 2829-2840.

Lewis, G.J., Panizzon, M.S., Eyler, L.T., Fennema-Notestine, C., Chen, C-H., Neale, M.C., Jernigan, T.L., Lyons, M.J., Dale, A.M., Kremen, W.S., & Franz, C.E. (2014). Heritable influences on amygdala and orbitofrontal cortex contribute to genetic variation in core dimensions of personality. *Neuroimage, 103*, 309-315.

Liu, W. Y., Weber, B., Reuter, M., Markett, S., Chu, W. C., & Montag, C. (2013). The big five of personality and structural imaging revisited: a VBM–DARTEL study. *Neuroreport, 24*, 375-380.

Lu, F., Huo, Y., Li, M., Chen, H., Liu, F., Wang, Y., ... & Chen, H. (2014). Relationship between personality and gray matter volume in healthy young adults: a voxel-based morphometric study. *PloS One*, *9*, e88763.

Nostro, A. D., Müller, V. I., Reid, A. T., & Eickhoff, S. B. (2017). Correlations between personality and brain structure: A crucial role of gender, *Cerebral Cortex, 27*, 3698-3712.

Riccelli, R., Toschi, N., Nigro, S., Terracciano, A., & Passamonti, L. (2017). Surface-based morphometry reveals the neuroanatomical basis of the five-factor model of personality. *Social Cognitive and Affective Neuroscience, 12*, 671-684.
